# One material, multiple functions: graphene/Ni(OH)_2_ thin films applied in batteries, electrochromism and sensors

**DOI:** 10.1038/srep33806

**Published:** 2016-09-22

**Authors:** Eduardo G. C. Neiva, Marcela M. Oliveira, Márcio F. Bergamini, Luiz H. Marcolino, Aldo J. G. Zarbin

**Affiliations:** 1 Departamento de Química, Universidade Federal do Paraná (UFPR), CP 19081, CEP 81531-990, Curitiba, P. R., Brazil; 2 Departamento de Química e Biologia, Universidade Tecnológica Federal do Paraná (UTFPR), Curitiba, P. R., Brazil

## Abstract

Different nanocomposites between reduced graphene oxide (rGO) and Ni(OH)_2_ nanoparticles were synthesized through modifications in the polyol method (starting from graphene oxide (GO) dispersion in ethylene glycol and nickel acetate), processed as thin films through the liquid-liquid interfacial route, homogeneously deposited over transparent electrodes and spectroscopically, microscopically and electrochemically characterized. The thin and transparent nanocomposite films (112 to 513 nm thickness, 62.6 to 19.9% transmittance at 550 nm) consist of α-Ni(OH)_2_ nanoparticles (mean diameter of 4.9 nm) homogeneously decorating the rGO sheets. As a control sample, neat Ni(OH)_2_ was prepared in the same way, consisting of porous nanoparticles with diameter ranging from 30 to 80 nm. The nanocomposite thin films present multifunctionality and they were applied as electrodes to alkaline batteries, as electrochromic material and as active component to electrochemical sensor to glycerol. In all the cases the nanocomposite films presented better performances when compared to the neat Ni(OH)_2_ nanoparticles, showing energy and power of 43.7 W h kg^−1^ and 4.8 kW kg^−1^ (8.24 A g^−1^) respectively, electrochromic efficiency reaching 70 cm^2^ C^−1^ and limit of detection as low as 15.4 ± 1.2 μmol L^−1^.

Nickel hydroxide is a multifunctional material widely used in many areas, such as batteries^1^,^2^, electrochromic devices^3^,^4^, sensors^5^,^6^,^7^, fuel cells^8^,^9^ and catalysis^10^. All these applications involve redox reactions between Ni(OH)_2_ and NiOOH, both of them having two stable polymorphs: the α and β phases for Ni(OH)_2_, the γ and β phases for NiOOH^11^. The differences between these structures are the space between the layers and their organization, in which the α-Ni(OH)_2_ and γ-NiOOH have the larger distances and are less organized than the β-Ni(OH)_2_ and β-NiOOH, as can be seen in the scheme presented in Supplementary Fig. S1 (Electronic Supplementary Material). These structural differences lead to different reactivities, where the α-Ni(OH)_2_ and γ-NiOOH species present the better electrochemical responses. However, these phases are usually converted to the β phases during successive cycles of redox process and in highly alkaline media, decreasing their performances^12^,^13^. The phase interconversion can be avoided through the interaction between Ni(OH)_2_ and graphene. The preparation of graphene/Ni(OH)_2_ nanocomposites also increases the conductivity of the final material (comparing with neat Ni(OH)_2_), as well as minimizes significantly the particle aggregation and growth^14^,^15^,^16^,^17^.

Among the different methodologies employed to obtain graphene-like materials, the chemical exfoliation of graphite presents low cost and high yield. One method is based on the oxidation of graphite, followed by its exfoliation and reduction, yielding the so-called reduced graphene oxide (rGO)^18^. The rGO has remaining oxygenated functional groups in its structure, which are very useful for the synthesis of nanocomposites, acting as nucleating sites^19^,^20^,^21^.

One interesting and growing application of graphene/nickel hydroxide nanocomposites is in the field of energy storage devices, as alkaline batteries^22^. When compared with the lithium-ion batteries, the alkaline batteries possess a low cost and are more safety^16^. A great challenge in the battery field is to find materials able to associate high energy with a high power^22^. Some approaches to overcome this challenge are the use of nanocomposites based on conducting materials (like graphene) with nanoparticles of the electroactive material. In addition, the method employed to process the material for the application is a key step, where thick films can damage the performance of the device, blocking the access of the inner electroactive sites and avoiding the use of high charge-discharge currents. Our research group developed a very effective route to deposit thin, transparent and homogeneous films of different unprocessable materials, as carbon nanotubes^23^,^24^, graphene^24^,^25^,^26^, metallic nanoparticles and different kind of nanocomposites^25^,^26^,^27^,^28^,^29^, in which the film is stabilized at a liquid-liquid interface, and easily deposited over different kind of ordinary substrates.

Besides its use in energy storage devices, Ni(OH)_2_ is also widely used as electrochromic material and electrochemical sensor for a several kind of analytes as glucose^5^, hydrogen peroxide^30^, methanol^31^, phosphate^32^, etc. The electrochromic properties of the Ni(OH)_2_ have been the focus of many studies^4^,^33^,^34^, because it is colorless in the reduced state and presents a deep brown color in the oxidized form, which allows its application in electronic devices displays, smart windows, glasses, among others^33^,^35^. In the same way for the batteries, the use of nanocomposites of Ni(OH)_2_ with graphene improves the performance of this material, increasing coloration efficiency and stability^35^. In which concerns its application as sensor, we demonstrate recently the use of Ni(OH)_2_-modified electrodes to detect glycerol, an important analyte in the pharmaceutical and biofuel fields^36^,^37^. However, to the best of our knowledge, there are no reports employing graphene/nickel hydroxide nanocomposite for this application.

Herein, we report a complete work based on different rGO/Ni(OH)_2_ nanocomposites, going from the synthesis till multiple-applications, passing by the fully characterization and by the processing as thin films. First, an original synthetic route is presented, in which both the components are obtained together in one step and one pot, starting from graphene oxide (GO) and nickel acetate as precursors. Afterwards, the deposition of these materials over different substrates as thin, transparent and homogeneous films through the liquid-liquid interfacial route is demonstrated. Further, the spectroscopic, microscopic and electrochemical characterization of these materials are discussed and the data correlated to their structure and morphology. Finally, the films were applied as electrodes for alkaline batteries, as electrochromic materials and as electrochemical sensors.

## Results and Discussion

### Structural and morphological characterization of the nanocomposites

A key point for the synthesis of Ni(OH)_2_ through the polyol method is the temperature of the reaction. Temperatures near the boiling point of the EG (198 °C) promote the formation of aldehyde, which will reduce the metal ions as demonstrate in the reaction (1) and (2), respectively^38^,^39^. This is an interesting way to produce graphene/metallic nickel nanocomposites, as will be demonstrated elsewhere. Otherwise, the polyalcohol generates alkoxides at slight lower temperatures, through the reaction with the acetate ions^40^, as represented by the reaction (3). The acetate ions react with water from the hydrated metal precursor, generating hydroxyl anions (reaction 4), which reacts with the Ni^2+^ cations to originate the Ni(OH)_2_. So, a rigorous control of temperature is necessary in order to obtain controlled samples. Also, the concentration of both OH^−^in solution^41^,^42^ and alkoxides^43^ generated according equation (3) affects directly the yield and the nature of the obtained Ni(OH)_2_.

The proposal for the nanocomposites described here involves both the synthesis of Ni(OH)_2_ and the reduction of the graphene oxide (GO) concomitantly. For that, the temperature needs to be high enough to reduce the GO (according similar steps as described in equations (1) and (2)), but not too high to reduce the metallic cations. As will be shown following, the temperature of 190 °C is ideal to achieve these requirements.


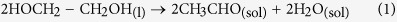



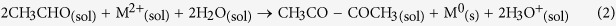



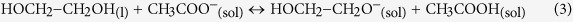



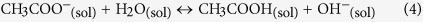


**Figure 1:**
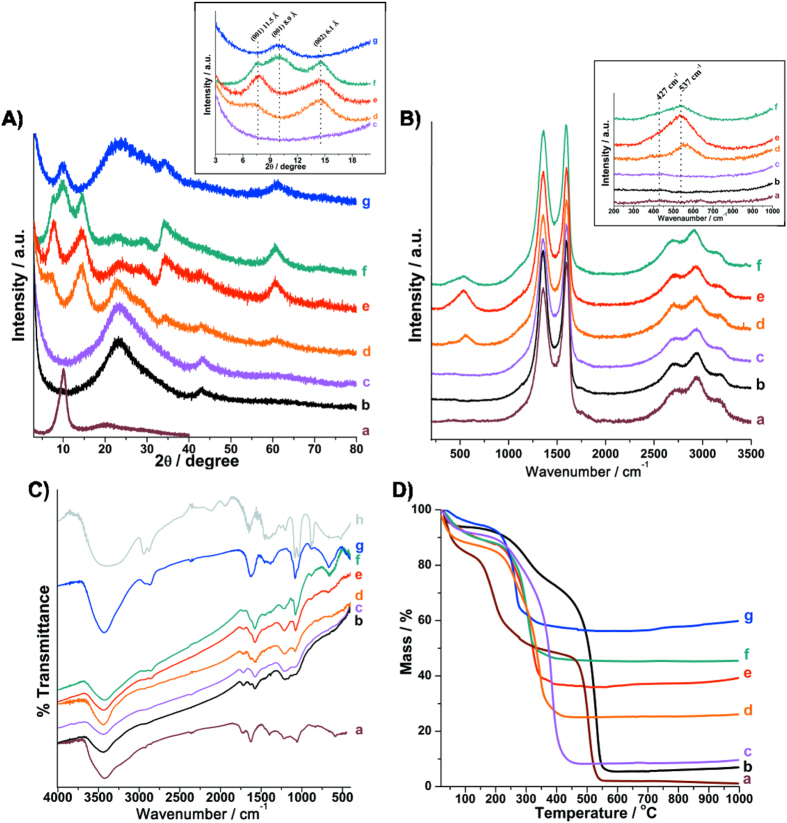
(**A**) X-ray diffractograms, (**B**) Raman spectra, (**C**) FT-IR spectra and (**D**) thermogravimetric curves of the samples **GO** (a), **rGO** (b), **rGONi(OH)**_**2**_**-1** (c), **rGONi(OH)**_**2**_**-2** (d), **rGONi(OH)**_**2**_**-3** (e), **rGONi(OH)**_**2**_**-4** (f), **Ni(OH)**_**2**_ (g) and ethylene glycol (h). Details of the low angle region of the XRD profiles and of the low wavenumber region of the Raman spectra are presented in the insets in (**A,B**), respectively. The TG data were obtained under air atmosphere at 5 °C min^−1^.

The X-ray diffraction patterns for all the nanocomposite samples, as well as for the **GO** (used as precursor), the **rGO** and the **Ni(OH)**_**2**_ (control samples) are shown in the Fig. 1A. The **GO** pattern (Fig. 1A-a) shows a sharp peak at d = 8.8 Å, due the (002) planes, characteristic of the interlayer separation in oxidized graphite. This peak disappears and another one at d = 3.8 Å is observed in the diffractograms of the **rGO** and of all the nanocomposites, indicating that the GO was reduced in all the samples. The XRD pattern of the **Ni(OH)**_**2**_ sample prepared as control (Fig. 1A-g) shows peaks at d = 8.9, 2.6 and 1.5 Å, characteristics of the (001), (100) and (110) planes of α-Ni(OH)_2_, respectively^33^,^44^.

The **rGONi(OH)**_**2**_**-2**, **rGONi(OH)**_**2**_**-3** and **rGONi(OH)**_**2**_**-4** diffractograms also exhibited the α-Ni(OH)_2_ peaks. However, a careful analysis of the XRD patterns of the nanocomposite samples demonstrates a different profile in the low angle region (inset of the Fig. 1A), when compared to the **Ni(OH)**_**2**_ sample. The **rGONi(OH)**_**2**_**-2** and the **rGONi(OH)**_**2**_**-3** possess a larger interlayer distance (11.5 Å) than the **Ni(OH)**_**2**_ (8.9 Å), and the **rGONi(OH)**_**2**_**-4** shows a mixture of the structures found in the **rGONi(OH)**_**2**_**-3** and in the **Ni(OH)**_**2**_. These different structural characteristics result from both the presence of the **GO** and the amount of nickel precursor used in the synthesis. The higher interlayer distances induced by the presence of **GO** in reactional medium are very interesting for electrochemical applications, since they would facilitate the access to the electroactive sites of the material, leading to a better performance in the desired application. The **rGONi(OH)**_**2**_**-1** did not showed peaks related to α-Ni(OH)_2_ due the smaller amount of nickel precursor used.

Figure 1B shows the Raman spectra of the samples. All the materials present the D (1355 cm^−1^), G (1582 cm^−1^), D’ (1600 cm^−1^), G’ (2699 cm^−1^), D + G (2937 cm^−1^) and 2D’ (3191 cm^−1^) bands characteristics of graphene-based materials obtaining by the chemical exfoliation of graphite^18^. The spectra of **rGONi(OH)**_**2**_**-2**, **rGONi(OH)**_**2**_**-3** and **rGONi(OH)**_**2**_**-4** show also bands at 427 and 537 cm^−1^ (inset of Fig. 1B), which are attributed to the Ni-OH and Ni-O^−^ stretching, respectively^44^. The last band is associated to structural defects generally found in the α-Ni(OH)_2_^45^,^46^.

The FT-IR spectra of the materials, compared to the neat ethylene glycol, are shown in the Fig. 1C. Bands at 3570/3425/3190 (ν_OH_), 2962/2920/2850 (ν_CH_), 1726 (ν_C=O_), 1625 (δ_H-O-H_), 1574 (ν_C=C_), 1402 (δ_C-OH_), 1220 (ν_C-O-C_) and 1060 cm^−1^ (ν_C-O_), all related to the presence of oxygenated groups at the surface, are still present in the rGO spectra, indicating that the reduction process converting the GO to rGO was not enough to eliminate all the oxygenated groups of the surface, as previously reported^18^. The FT-IR spectra of the nanocomposites and of the control sample **Ni(OH)**_**2**_ also show bands at 665 and 586 cm^−1^, ascribed to δ_Ni(OH)2_ and ν_Ni(OH)2_, respectively^46^,^47^, as well as bands at 2939 (antisymmetric ν _C-H_), 2878 (symmetric ν_C-H_), 1458 (δ_C-H_), 1408 (δ_C-O-H_), 1323 (γ_C-H2_), 1086 (symmetric ν_C-O_), 1039 (antisymmetric ν_C-O_) and 865 cm^−1^ (ν_C-C_)^48^, related to ethylene glycol, indicating that the polyalcohol is present in the final material. As higher is the **Ni(OH)**_**2**_ amount in the samples, the higher the relative intensity of the ethylene glycol bands. This data suggest that the ethylene glycol is probably acting as stabilizer of the Ni(OH)_2_ nanoparticles, as we have previously observed for neat metallic Ni nanoparticles produced by the polyol route^49^.

Figure 1D shows the thermogravimetric (TG) curves of the samples, collected under air atmosphere. Comparing the TG curves of **GO** and **rGO**, the mass loss event due to the release of the oxygenate groups (120 to 400 °C) decreases from **GO** (36.6%) to **rGO** (20.9%), leading to a change of the C/O rate from 1.25 to 3.11. The TG curve of **rGO** also presents an increase in the oxidation temperature of the carbon backbone of 27 °C in comparison to the GO, due the restoration of the sp^2^ C-C bonds, as also observed in the DTG curves showed in the Supplementary Fig. S2A. Regarding to the nanocomposites, there is an increase of the residue amount as the higher the mass of nickel acetate initially used, as expected. Taking into account that the residue is composed by NiO, the Ni(OH)_2_ amount in the initial samples were calculate and the values are presented in the Supplementary Table S1. The Ni(OH)_2_ percentage increases, as expected, from 11.9 (**rGONi(OH)**_**2**_**-1**) to 56.4% (**rGONi(OH)**_**2**_**-4**). The ratio between Ni(OH)_2_ and rGO in the sample is also important for electrochemical applications, since excess of rGO can block the access to the Ni(OH)_2_ nanoparticles. There is also a decrease of the oxidation temperature of the carbon backbone with the increase of the Ni(OH)_2_ content in the sample, which acts as hot spots catalyzing the carbon degradation. As observed in the Supplementary Table S1, the weight loss of the first event related to the release of intercalated water is higher for the nanocomposites than for the neat **Ni(OH)**_**2**_, confirming the larger layer spacing of the α-Ni(OH)_2_ nanoparticles in the nanocomposites. The oxidation temperatures obtained from the DTG curves (Supplementary Fig. S2A) described in Supplementary Table S1 are very close to the oxidation peaks observed in the DSC curves (Supplementary Fig. S2B).

**Figure 2:**
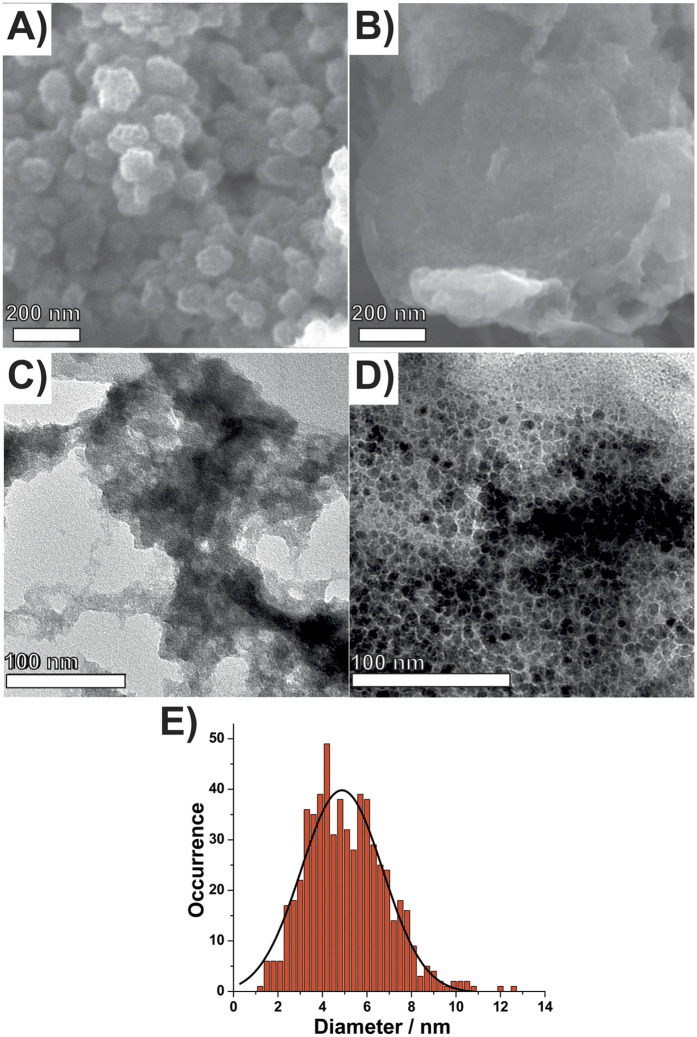
(**A,B**) FEG-SEM and (**C,D**) TEM images of (**A,C**) control **Ni(OH)**_**2**_ and (**B,D**) **rGONi(OH)**_**2**_**-4**. (**E**) Size distribution histogram for the Ni(OH)_2_ nanoparticles on **rGONi(OH)**_**2**_**-4**.

Figure 2A,B shows the FEG-SEM images of neat **Ni(OH)**_**2**_ and **rGONi(OH)**_**2**_**-4**, respectively. The **Ni(OH)**_**2**_ sample is composed by porous spherical particles with size ranging from 30 to 80 nm. In contrast, the **rGONi(OH)**_**2**_**-4** is composed of rGO sheets decorated with many nanoparticles. Regarding to the nanocomposites with smaller Ni(OH)_2_ percentages (Supplementary Fig. S3), the Ni(OH)_2_ nanoparticles were not detectable. The TEM images of the **Ni(OH)**_**2**_ and **rGONi(OH)**_**2**_**-4** are presented in the Fig. 2C,D, respectively. As seen in the FEG-SEM images, the **Ni(OH)**_**2**_ possesses a porous structure like a foam and the **rGONi(OH)**_**2**_**-4** exhibits rGO sheets highly decorated by Ni(OH)_2_ nanoparticles. Size histogram for these nanoparticles plotted in the Fig. 2E shows a narrow size distribution with a mean size of 4.9 ± 1.8 nm. In the same way as **rGONi(OH)**_**2**_**-4**, **rGONi(OH)**_**2**_**-3** exhibits the Ni(OH)_2_ nanoparticles decorating the rGO sheets, however, with fewer nanoparticles, as showed in the Supplementary Fig. S4. These data prove the GO has an important role in the Ni(OH)_2_ synthesis, affecting the structure and the morphology of the Ni(OH)_2_ nanoparticles.

The EDS spectra of the materials are shown in the Supplementary Fig. S5A. As observed in TG curves, as the higher the nickel acetate amount initially used as precursor in the synthesis, the higher the nickel and oxygen peaks intensities. The control **Ni(OH)**_**2**_ spectra also presents a peak related to carbon, confirming the presence of the ethylene glycol in the samples. Based on these spectra, the nickel/oxygen, nickel/carbon and oxygen/carbon peak areas ratios were calculated (Supplementary Fig. S5B). As expected, there is an increase of the nickel/carbon and oxygen/carbon peak areas ratios as the higher the Ni(OH)_2_ percentage in the samples.

### Thin films characterization

**Figure 3:**
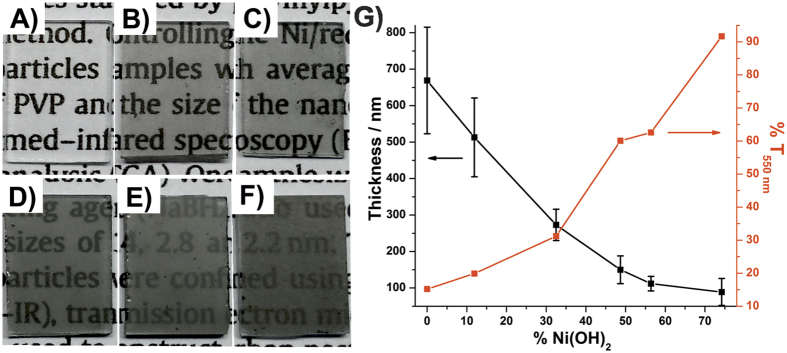
(**A**) Photographic images of **Ni(OH)**_**2**_, (**B**) **rGONi(OH)**_**2**_**-4**, (**C**) **rGONi(OH)**_**2**_**-3**, (**D**) **rGONi(OH)**_**2**_**-2**, (**E**) **rGONi(OH)**_**2**_**-1** and (**F**) **rGO** thin films over glass substrates. (**G**) Thickness and transmittance at 550 nm as function of Ni(OH)_2_ percentage in the samples.

The photographic images of the thin films prepared by the biphasic system are shown in the Fig. 3A–F. It is noted that the thin films are homogeneous and possess different transmittance values. As the rGO percentage increase in the materials, the transmittance of the thin films decreases. This behavior is due an increase of the thickness of the thin films with the rGO percentage, from 112 ± 20 (**rGONi(OH)**_**2**_**-4**) to 513 ± 108 nm (**rGONi(OH)**_**2**_**-1**), resulting in a change in the transmittance from 62.6 (**rGONi(OH)**_**2**_**-4**) to 19.9% (**rGONi(OH)**_**2**_**-1**), as exposed in the Fig. 3G. Taking into account that the rGO and Ni(OH)_2_ have different densities and it was used the same mass of the materials (1 mg) to prepare the thin films, as the higher the rGO percentage in the nanocomposite, the higher the volume the thin film will occupy in the liquid-liquid interface, increasing the thickness of the film over the substrate. As commented before, thicker films are not desirable, as **rGONi(OH)**_**2**_**-1** and **rGONi(OH)**_**2**_**-2** nanocomposites, damaging the electrochemical and electrochromic properties of the film. The UV-Vis spectra in the absorbance mode of the thin films are shown in the Supplementary Fig. S6. The spectra of the nanocomposites and the rGO thin films show typical graphene profile with a band at 270 nm attributed to the π-π* transition.

As the nanocomposites are composed of graphene-based materials, the sheet resistance of the thin films were measured over glass substrates and the values were 754 ± 33, 504 ± 50, 2800 ± 450 and 53000 ± 9500 kΩ □^−1^ for **rGO**, **rGONi(OH)**_**2**_**-1**, **rGONi(OH)**_**2**_**-2** and **rGONi(OH)**_**2**_**-3**, respectively. The high values observed are due the synthetic method used, which does not eliminate completely the oxygenate groups of the GO, affecting the conductivity of the rGO. The increase of the resistivity with the Ni(OH)_2_ content happens because the Ni(OH)_2_ shows a poor conductivity. Due the **rGONi(OH)**_**2**_**-4** and obviously the control **Ni(OH)**_**2**_ present the highest Ni(OH)_2_ content, leading to the highest sheet resistance values, the absolute values of the sheet resistance of these films were unable to be measured by the equipment.

Supplementary Fig. S7A–C show the FEG-SEM images of the control **Ni(OH)**_**2**_ and **rGONi(OH)**_**2**_**-4** thin films over ITO substrates, respectively. As observed in the FEG-SEM image of the powder (Fig. 2A), the **Ni(OH)**_**2**_ thin film is composed by spherical nanoparticles, where some of them are isolated and others in large agglomerates. In contrast, besides the rGO sheets covering the substrate, the **rGONi(OH)**_**2**_**-4** thin film does not exhibit large agglomerates, what is an advantage for electrochemical applications. The same morphology was found to the others nanocomposites. The nature of these nanoparticles as being Ni(OH)_2_ was confirmed by EDS, where the punctual EDS spectra over the nanoparticles exhibits the nickel signal and an increase in the oxygen signal intensity (Supplementary Fig. S8).

### Electrochemical characterization of the thin films in alkaline medium

**Figure 4:**
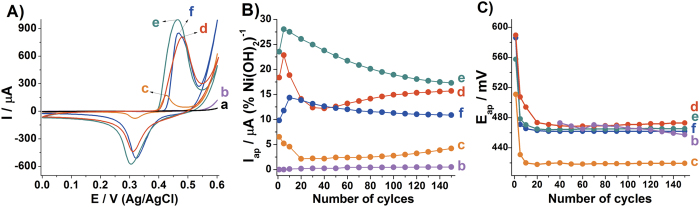
(**A**) 150^th^ cyclic voltammograms, (**B**) anodic peak current normalized by the Ni(OH)_2_ percentage in each sample and (**C**) anodic peak potential as function of cycling of **rGO** (a), **rGONi(OH)**_**2**_**-1** (b), **rGONi(OH)**_**2**_**-2** (c), **rGONi(OH)**_**2**_**-3** (d), **rGONi(OH)**_**2**_**-4** (e) and **Ni(OH)**_**2**_ (f) in aqueous solution of NaOH 1 mol L^−1^ at 50 mV s^−1^.

The 150^th^ cyclic voltammograms of the thin films in 1 mol L^−1^ NaOH aqueous medium are presented in the Fig. 4A. With exception of the control **rGO** thin film, all the electrodes exhibited the Ni(OH)_2_/NiOOH redox pair and as the higher the Ni(OH)_2_ content the higher the current peak intensities. However, the normalized current peak intensities by the Ni(OH)_2_ percentage are higher for the nanocomposites **rGONi(OH)**_**2**_**-4** and **rGONi(OH)**_**2**_**-3** than the **Ni(OH)**_**2**_, as can be observed in the Fig. 4B. This behavior is resulted from the differences between the structure and morphologies of the Ni(OH)_2_ nanoparticles and the thin films for each sample. As the **Ni(OH)**_**2**_ control sample possess a smaller interlayer distance, a bigger nanoparticles size and the thin film possess big agglomerates, it is expected that this sample should present the lower current response. It is also noted in the Fig. 4B that the anodic current peak intensities decrease after the first cycles for the samples **rGONi(OH)**_**2**_**-4** and neat **Ni(OH)**_**2**_, which can be due an increase of the nanoparticle size and/or of the crystallinity. For the **rGONi(OH)**_**2**_**-3**, **rGONi(OH)**_**2**_**-2** and **rGONi(OH)**_**2**_**-1** thin films, there is an increase of anodic current after the first cycle. This suggest that initially the higher rGO percentage in these films blocks the access to the Ni(OH)_2_ nanoparticles, and as the thin films starts to be cycled these nanoparticles became exposed. This behavior also explains the lower current peak intensities even though when normalized by the Ni(OH)_2_ percentage. Regarding to the peak potential, after the first cycle the peak potential stabilizes for all the thin films, indicating no significant crystallinity changes.

After the 150 cyclic voltammograms the thin films were analyzed by FEG-SEM and presented the same morphology as before cycling. However, the thin films with lower Ni(OH)_2_ content exhibited a higher number of nanoparticles than before cycling (Supplementary Fig. S9), confirming the exposition of these nanoparticles with the cycling as indicated by the voltammetric data.

Supplementary Fig. S10A,B show the X-ray diffraction patterns of the thin films before and after 150 voltammetric cycles. As can be seen, only the control **Ni(OH)**_**2**_ thin film exhibits the peak at 10° related to the (001) planes of α-Ni(OH)_2_ before the cycling. After the cycling, this peak shifts to 12° indicating a slightly change in the interlayer space of Ni(OH)_2_ nanoparticles (from 8.8 to 7.4 Å). The nanocomposites did not exhibit this peak before the cycling, which can be due to the lower amount of the Ni(OH)_2_ in them and the covering of the Ni(OH)_2_ nanoparticles by the rGO sheets. The nanocomposites and **rGO** thin films also present a peak at about 23° in 2θ related to rGO and it is more evident as the higher the rGO content. As seen for the **Ni(OH)**_**2**_control sample, the **rGONi(OH)**_**2**_**-4** and **rGONi(OH)**_**2**_**-3** thin films show a peak at 12° attributed to the (001) of α-Ni(OH)_2_ after the 150^th^ cycle, indicating a decrease of the interlayer distance from 11.5 and 8.9 Å in the powder samples to 7.4 Å. Although the nanocomposites and the **Ni(OH)**_**2**_ control sample showed an increase in the crystallinity of the nanoparticles, they still present the α phase and it is an important characteristic for the further applications.

The influence of the scan rate in the Ni(OH)_2_/NiOOH redox pair after the pre-treatment of 150 cyclic voltammograms at 50 mV s^−1^ was evaluated, Supplementary Fig. S11. For all the thin films is observed a linear dependence of the current peak intensities with the root square of the scan rate (v^1/2^), suggesting a diffusion-controlled process for the electroactive species involved in the Ni(OH)_2_/NiOOH redox pair^36^.

### Application as electrodes for alkaline and Li ion batteries

**Figure 5:**
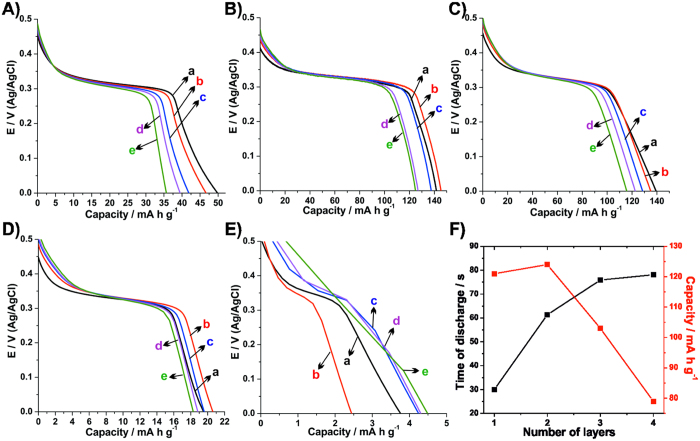
Discharge curves: (**A**) control **Ni(OH)**_**2**_, (**B**) **rGONi(OH)**_**2**_**-4**, (**C**) **rGONi(OH)**_**2**_**-3**, (**D**) **rGONi(OH)**_**2**_**-2** and (**E**) **rGONi(OH)**_**2**_**-1** thin films using 0.82 (a), 1.65 (b), 3.29 (c), 4.94 (d) and 8.24 A g^−1^ (e). (**F**) Time of discharge and capacity as function of the number of layers to the **rGONi(OH)**_**2**_**-4** thin film using a charge-discharge current of 8.24 A g^−1^.

The potentiality of the thin films as electroactive materials for alkaline batteries was evaluated and the discharge curves obtaining after the voltammetric pre-treatment are presented in the Fig. 5. The capacity (C) of the thin films was calculated using the equation (5),


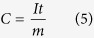


where I is the charge-discharge current (mA), t is the time of discharge (h) and m is the mass of material on the electrode (g), in which only the mass of the electroactive specie Ni(OH)_2_ was considered. As can be seen in Fig. 5, all the thin films exhibit a plateau in the range from 0.3 to 0.4 V related to the reduction of NiOOH. This behavior is typical of batteries in which the energy storage is due faradaic processes^22^. It is also observed that the increase of the charge-discharge current leads to a slight decrease of the capacity. This usually happens in batteries due the need of electroactive species involved in the faradaic processes diffuse through the film, and as bigger the particle size and the thickness of the film, the higher is the capacity losses. The **rGONi(OH)**_**2**_**-1** did not exhibit this decrease in the capacity with the increase of the charge-discharge current, which can be related to the exposition of the Ni(OH)_2_ nanoparticles with the cycling, leading to an increase in the charge stored in the material. Comparing the discharges curves, the **rGONi(OH)**_**2**_**-4** and **rGONi(OH)**_**2**_**-3** presented the higher capacities, as expected since they presented the higher voltammetric responses.

The liquid-liquid interfacial route can be easily used to produce films with several superposed layers, which is an advantage for the construction of final energy storage device and leads to a higher charge stored. Therefore, the influence of the number of layers on the time of discharge and the capacity for the **rGONi(OH)**_**2**_**-4** nanocomposite was evaluated (Fig. 5F). It is clear a proportional increase on the time of discharge after the deposition of a second layer, maintaining the capacity. For the third layer there is a non-proportional increase on the time of discharge, and for the fourth layer no significant increase has been detected, followed by a decrease in the capacity, probably due the covering of the inner layers by the rGO sheets, blocking the access to the Ni(OH)_2_ nanoparticles. As the time of discharge still increase till the deposition of three layers thin film, this could be considered the ideal configuration. The photographic images of thin films varying the number of layers of **rGONi(OH)**_**2**_**-4**, as well as the ITO are present in the Supplementary Fig. S12A. As expected, the increase of the number of layers leads to a decrease in the transmittance from 75 to 38% at 550 nm (Supplementary Fig. S12B).

**Figure 6:**
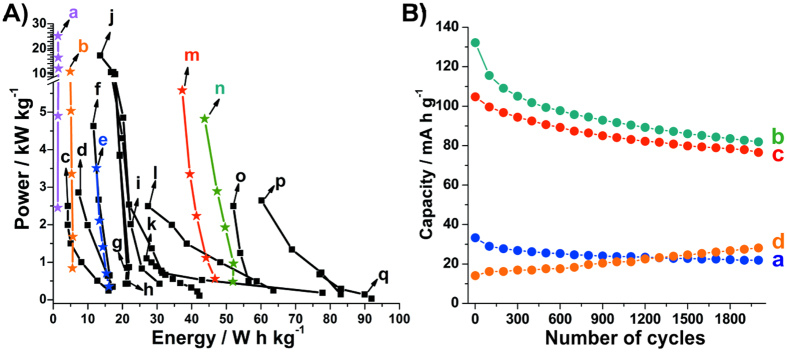
(**A**) Specific power as function of the specific energy (Ragone’s plot) for the **rGONi(OH)**_**2**_**-1** (a), **rGONi(OH)**_**2**_**-2** (b), **rGONi(OH)**_**2**_**-3** (m), **rGONi(OH)**_**2**_**-4** (n), control **Ni(OH)**_**2**_ (e) and for reports from literature using Ni(OH)_2_ nanoparticles (c^55^, o^56^, q)^1^, activated carbon-NiO-Ni(OH)_2_ (d)^57^, rGO-NiO-Ni(OH)_2_ (f)^57^, MWCNT-NiO-Ni(OH)_2_ (g)^57^, SWCNT-NiO-Ni(OH)_2_ (h)^57^, rGO-Ni(OH)_2_ nanocomposites (i^58^, j^59^, l^56^, p)^16^ and aluminum-substituted Ni(OH)_2_ (k)^60^. (**B**) Capacity as function of cycling for control **Ni(OH)**_**2**_ (a), **rGONi(OH)**_**2**_**-4** (b), **rGONi(OH)**_**2**_**-3** (c) and **rGONi(OH)**_**2**_**-2** (d) using 8.24 A g^−1^.

The specific energy (E) and power (P) values for the thin films were calculated using the equations (6) and (7), respectively, where C is the capacity (mA h g^−1^), V is the plateau voltage (V) and t is the time of discharge (h). The obtained values for the thin films prepared in this work compared with other data involving Ni(OH)_2_ nanoparticles and nanocomposites with different carbonaceous materials are demonstrated in the Rogone’s plot present in Fig. 6A. As can be seen, the **rGONi(OH)**_**2**_**-4** and **rGONi(OH)**_**2**_**-3** exhibited the higher specific energy and power values among the thin films developed in this work, and impressive values comparing with the literature (Fig. 6A). Other important characteristic is the high energy values even though when using high charge-discharge currents, demonstrating their potentiality for fast energy devices.


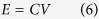



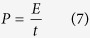


Figure 6B shows the stability of the capacity applying 2000 charge-discharge cycles using a current of 8.24 A g^−1^ for the nanocomposites and for the neat **Ni(OH)**_**2**_ thin films. Except for the **rGONi(OH)**_**2**_**-2**, all the thin films presented a decrease in the capacity with the exhaustive cycling, where the **rGONi(OH)**_**2**_**-3**, **rGONi(OH)**_**2**_**-4** and control **Ni(OH)**_**2**_ exhibited 73, 62 and 66% of the initial capacity, respectively. This loss of capacity can be attributed to structural and morphological changes in the Ni(OH)_2_ nanoparticles, decreasing the number of electroactive sites. The increase in the capacity of the **rGONi(OH)**_**2**_**-2** (200% of the initial capacity) is related to the exposition of the Ni(OH)_2_ nanoparticles with the cycling, as discussed before. These structural and morphological changes also affected the voltammetric behavior and the EIS spectra of the thin films, as can be seen in Supplementary Figs S13 and S14, respectively. As seen in the Supplementary Fig. S13, the **rGONi(OH)**_**2**_**-3**, **rGONi(OH)**_**2**_**-4** and control **Ni(OH)**_**2**_ presented a shift and an intensity decrease of the peaks. The EIS spectra of the thin films before the charge-discharge cycling present semicircles in the high-frequency range, which are related to the oxidation process of the Ni(OH)_2_ (Supplementary Fig. S14A,B). The curves were fitted using the equivalent circuit illustrated in the inset of the Supplementary Fig. S14B. R_b_ is the resistance resulting from the contact resistance between the nanomaterial and the current collector, the intrinsic resistance of the substrate and the electrolyte resistance; CPE is the constant phase element and represents the double-layer capacitance; R_ct_ is the charge-transfer resistance resulting from the faradaic reaction; Z_w_ is the Warburg element related to the ion diffusion resistance; and C_int_ is the capacitance originated from the ion intercalation in the Ni(OH)_2_ layers^50^,^51^. The values of R_ct_ to **rGONi(OH)**_**2**_**-3**, **rGONi(OH)**_**2**_**-4** and control **Ni(OH)**_**2**_ thin films before the charge-discharge cycling were 30, 28 and 23 Ω, respectively. The low values of R_ct_ are resulting of the high surface area of the Ni(OH)_2_ nanoparticles^50^,^51^. However, after the 2000 charge-discharge cycles there is a drastic change in the EIS profile for the three thin films, as seen in the Supplementary Fig. S14C. This high increase of the semicircle length indicates a very high R_ct_ and it is also associated to structural and morphological changes after this long cycling. Due the very high R_ct_, the EIS spectra after charge-discharge cycling were not fitted.

To evaluate possible changes in the Ni(OH)_2_ structure, the thin films were analyzed by X-ray diffraction before and after the 2000 charge-discharge cycles (Supplementary Fig. S15). Both patterns present the broad peak at around 11° (001) related to the α-Ni(OH)_2_. Nevertheless, the thin films after the charge-discharge cycling also exhibit a broad and low intensity peak at around 21° (001), which could be related to a defective β-Ni(OH)_2_ formed in the films^44^. This slight change in the crystallinity of the α-Ni(OH)_2_ indicate that probably morphological changes as particle agglomeration happened after the 2000 charge-discharge cycles and they should be responsible by the decrease of the capacity.

Aiming evaluate the potentiality of the nanocomposites as electroactive materials for Li-ion batteries, the **rGONi(OH)**_**2**_**-4** thin film was cycled in LiClO_4_/propylenecarbonate solution. Supplementary Fig. S16 shows the second voltammetric cycle using a scan rate of 1 mV s^−1^. It is clear to note the presence of two anodic and two cathodic peaks. As describe by others^52^,^53^, the reduction processes are related to both the formation of the solid electrolyte interface (SEI) film on the nanocomposite electrode (at −0.408 V, which consist of several lithium species as Li_2_CO_3_ and RCO_2_Li)^54^, and the reduction of the Ni(OH)_2_ to Ni (at −0.062 V, with consequent production of LiOH). In opposite, the anodic processes are attributed to the decomposition of the SEI (at 1.640 V) and the oxidation of the metallic Ni (at 1.374 V, with consequent decomposition of the LiOH). In this way, the lithium intercalation process can be described as:


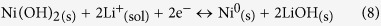


This result indicates the potentiality of use the rGONi(OH)_2_ nanocomposites for this purpose.

### Application as electrochromic material in alkaline medium

**Figure 7:**
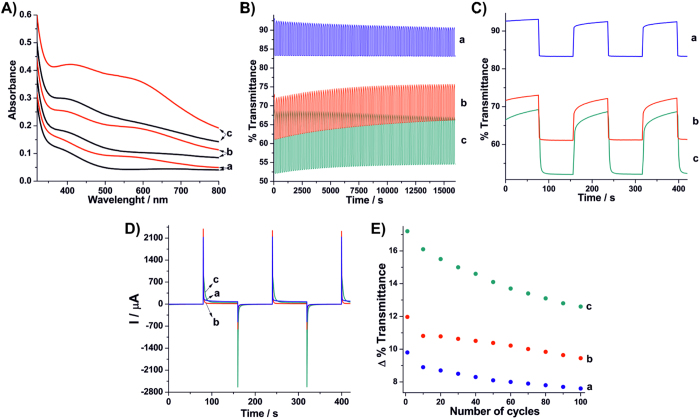
(**A**) UV-Vis spectra applying 0.1 (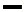) and 0.5 V (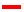), (**B,C**) transmittance as function of the time for the 100 colored-bleached cycles, (**D**) chronoamperograms for the potentials applied and (**E**) transmittance variation as function of the number of cycles for the control **Ni(OH)**_**2**_ (a), **rGONi(OH)**_**2**_**-3** (b) and **rGONi(OH)**_**2**_**-4** (c) thin films.

Since the **rGONi(OH)**_**2**_**-4**, **rGONi(OH)**_**2**_**-3** and control **Ni(OH)**_**2**_thin films have the higher transmittances, they were also evaluated as electrochromic electrodes. For this purpose, after the pre-treatment of 150 voltammetric cycles, the thin films were periodically submitted to potentials of 0.1 and 0.5 V and simultaneously analyzed by UV-Vis spectroscopy. As seen in the Fig. 7A, the change of the applied potential from 0.1 to 0.5 V leads to an increase of the absorbance. This happens because the NiOOH generated at 0.5 V is darker than the Ni(OH)_2_ generated at 0.1 V. However, the thin films presented different absorbance variations (optical densities), where the values at 550 nm were 0.047, 0.154 and 0.090 to the **Ni(OH)**_**2**_, **rGONi(OH)**_**2**_**-4** and **rGONi(OH)**_**2**_**-3** thin films, respectively. In the same way that observed during the data related to the application in batteries, the nanocomposite thin films present a better response than the neat **Ni(OH)**_**2**_. Figure 7B shows the transmittance at 550 nm of the thin films as function of time, where 100 colored-bleached cycles were applied. As can be seen in the UV-Vis spectra (Fig. 7A), the **rGONi(OH)**_**2**_**-4** thin film exhibited the higher transmittance variation (Fig. 7C), which is due the higher percentage of Ni(OH)_2_ and the smaller particle size comparing with the **rGONi(OH)**_**2**_**-3** and control **Ni(OH)**_**2**_, respectively. The response time was considered as the time to reach 2/3 of the total transmittance change. The obtained values were 3.9, 1.7 and 1.7 s to the **rGONi(OH)**_**2**_**-4**, **rGONi(OH)**_**2**_**-3** and control **Ni(OH)**_**2**_ thin films, respectively. The higher response time to the **rGONi(OH)**_**2**_**-4** can be related to the higher amount of particles decorating the rGO. Taking into account the optical density (OD) and the charge (Q) of the last colored-bleached cycle, and using the equation (9), the electrochromic efficiency (η) for the **rGONi(OH)**_**2**_**-4**, **rGONi(OH)**_**2**_**-3** and control **Ni(OH)**_**2**_ thin films were 52, 70 and 58 cm^2^ C^−1^, respectively. Once more the nanocomposites exhibited a better performance than the neat **Ni(OH)**_**2**_. These η values are very close to the results of literature reports that use Ni(OH)_2_ nanoparticles (80 cm^2^ C^−1^)^34^, Ni(OH)_2_ thin films (111 cm^2^ C^−1^)^4^ and rGO/Ni(OH)_2_ nanocomposites (76 cm^2^ C^−1^)^35^.

Figure 7E presents the stability of the transmittance variation as function of the cycling. All the thin films presented a decrease of the transmittance variation with the cycling, reaching to 73.3, 79 and 77.6% of initial value for the **rGONi(OH)**_**2**_**-4**, **rGONi(OH)**_**2**_**-3** and control **Ni(OH)**_**2**_, respectively. This decrease is expected, since morphological changes in the Ni(OH)_2_ nanoparticles happen with the cycling of the materials.


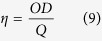


### Application as chronoamperometric sensor for glycerol

**Figure 8:**
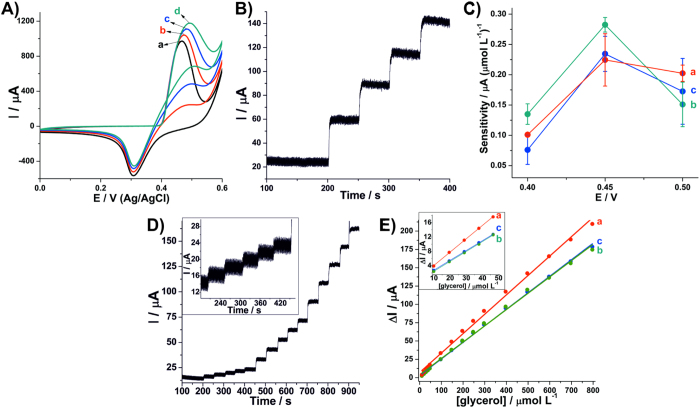
(**A**) Cyclic voltammograms of **rGONi(OH)**_**2**_**-4** thin film in the absence (a) and presence of 1 (b), 2 (c) and 3 (d) mmol L^−1^ glycerol. (**B**) Chronoamperogram for **rGONi(OH)**_**2**_**-4** thin film with four additions of 100 μmol L^−1^ glycerol using 0.45 V. (**C**) Sensitivity as function of the potential used in the chronoamperometry. (**D**) Chronoamperogram for **rGONi(OH)**_**2**_**-4** thin film with glycerol additions from 10 to 800 μmol L^−1^. (**E**) Analytical curves constructed from chronoamperograms. (a–c)in (**C**,**E**) represent **rGONi(OH)**_**2**_**-3** (a), **rGONi(OH)**_**2**_**-4** (b) and control **Ni(OH)**_**2**_(c) thin films. The analyses were carried out in 1 mol L^−1^ NaOH aqueous solution.

Ni(OH)_2_ is also widely used as electroactive material for electrochemical sensors. For this reason, the **rGONi(OH)**_**2**_**-4**, **rGONi(OH)**_**2**_**-3** and control **Ni(OH)**_**2**_ thin films were evaluated as sensors for glycerol. For that, after the pre-treatment of 150 voltammetric cycles, three additions of 1 mmol L^−1^ of glycerol have been done to the electrochemical cell and after each addition the electrodes were cycled. Figure 8A shows the voltammograms for the **rGONi(OH)**_**2**_**-4** thin film in the absence and in the presence of glycerol. Both the increase of the anodic current peak and the decrease of the cathodic current peak indicate a typical electrocatalysis profile, where the NiOOH oxidizes the analyte generating Ni(OH)_2_ and products. Since the potential that this chemical step occurs is high, the resulting Ni(OH)_2_ is reoxidized leading to the current increment. Concomitantly, as the NiOOH is consumed the cathodic current peak decreases. The same behavior was found to the other thin films (Supplementary Fig. S17) and confirms the electroactivity of these nanomaterials for detection of glycerol. However, the **rGONi(OH)**_**2**_**-3** exhibits an expressive decrease of the cathodic current, indicating a higher electrocatalytic behavior.

To optimize the potential for the glycerol detection, the thin films were submitted to chronoamperometry using the potentials of 0.4, 0.45 and 0.5 V and for each potential four additions of 100 μmol L^−1^ glycerol were carried out (Supplementary Fig. S18). Figure 8B shows a typical chronoamperogram, showing the increases of the current according the glycerol additions, as expected. Using the current increments after each glycerol addition, the sensitivity for the thin films in each potential evaluated was calculated (Fig. 8C), and the best sensitivity for all the thin films was found at 0.45 V. So, the potential of 0.45 V was employed for the detection of glycerol in the concentration range from 10 to 800 μmol L^−1^. All the thin films showed a similar chronoamperogram (Fig. 8D and Supplementary Fig. S19) characterized by current increments for all the glycerol additions. Figure 8E exhibits the analytical curves constructed from the chronoamperograms. It is clear to note the linear responses for the whole glycerol concentration range, with R^2^ of 0.9987, 0.9978 and 0.9961 to the control **Ni(OH)**_**2**_, **rGONi(OH)**_**2**_**-4** and **rGONi(OH)**_**2**_**-3** thin films, respectively. However, the **rGONi(OH)**_**2**_**-3** leads to the higher sensitivity, where the values found to the concentration range from 10 to 50 μmol L^−1^ were 0.28 ± 0.01, 0.24 ± 0.06 and 0.36 ± 0.02 μA (μmol L^−1^)^−1^ to control **Ni(OH)**_**2**_, **rGONi(OH)**_**2**_**-4** and **rGONi(OH)**_**2**_**-3**, respectively. The limit of detection (LOD) and the limit of quantification (LOQ) were calculated using the Equations (10) and (11), respectively, where s is the standard deviation from the blank (1.9, 2.0 and 1.9 μA to **Ni(OH)**_**2**_, **rGONi(OH)**_**2**_**-4** and **rGONi(OH)**_**2**_**-3**, respectively), and α is the sensitivity. The **rGONi(OH)**_**2**_**-3** thin film presented the lower LOD and LOQ (15.4 ± 1.2 and 51.3 ± 4.1 μmol L^−1^), than the **Ni(OH)**_**2**_ (20.2 ± 0.6 and 67.5 ± 2.1 μmol L^−1^) and the **rGONi(OH)**_**2**_**-4** thin films (25.0 ± 3.1 and 83.2 ± 10.5 μmol L^−1^). Despite the **Ni(OH)**_**2**_ control sample showed a better performance than the **rGONi(OH)**_**2**_**-4**, it is important to notice that this last one possesses a lower amount of electroactive material. The higher electroactivity of the **rGONi(OH)**_**2**_**-3** can be associated to smaller nanoparticles sizes and a better distribution of them through the film. These low LOD values and the wide linear detection range (10 to 800 μmol L^−1^) reveal a good performance of the thin films developed in this work when compared with the literature, where LOD values vary from 1.49 to 95 μmol L^−1^, as seen in the Supplementary Table S2.


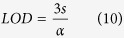



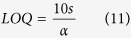


## Conclusions

Different nanocomposites between reduced graphene oxide and Ni(OH)_2_ nanoparticles have been synthesized by an innovative modification in the polyol method, starting from both graphene oxide and nickel acetate as precursors, in one pot and one single step. Comparing to neat Ni(OH)_2_ nanoparticles obtained by the same route, the hydroxide nanoparticles obtained in the nanocomposites are smaller and present a more open structure, which are very desirable characteristics for electrochemical applications. The concomitant occurrence of both the synthesis of Ni(OH)_2_ and the reduction of GO represent a new pathway for both reactions, in which the oxygenates groups of the GO surface can act as nucleating points for the Ni(OH)_2_ nanoparticles growth, resulting in nanocomposites with a strong interaction between the components.

All the materials prepared were processed as thin, homogenous and transparent films through a liquid-liquid biphasic system, and homogeneously deposited over transparent electrodes. These thin films presented a multifunctional character and they were applied as electrodes for alkaline batteries, electrochromic materials and active component in electrochemical sensors. For all these applications the performances of the nanocomposites were much superior than the observed for neat Ni(OH)_2_ prepared as control sample, which is attributed to: i) the smaller Ni(OH)_2_ particle size; ii) the better Ni(OH)_2_ distribution through the film; and iii) the presence of the rGO in the nanocomposites. Also, the performance for each application is better or comparable to the best results found in literature, with a particular emphasis to the application as electrodes for alkaline batteries, in which the responses of the nanocomposite films occupy a privileged position in the Ragone’s plot due the impressive and desirable relationship between the specific energy and power of the materials.

## Experimental

H_2_SO_4_ (Carlo Erba), graphite (Graflake 99580, 99%, Nacional de Grafite SA), NaNO_3_ (Vetec), KMnO_4_ (Synth), HCl (Impex), H_2_O_2_ aqueous solution (Vetec), ethylene glycol (Carlo Erba), toluene (Carlo Erba), glycerol (CAAL) and NaOH (Vetec) were used as received. Ni(CH_3_COO)_2_·4H_2_O (Vetec) was previously dried under low pressure at 100 ^◦^C for 5 hours before used. The aqueous solutions were prepared using deionized Milli-Q water.

The **GO** was obtained as follows^18^: 46 mL of concentrated H_2_SO_4_ was added to a 500 mL round flask under ice bath. Next, 2 g of graphite and 1 g of NaNO_3_ were added to the round flask. After 15 minutes of magnetic stirring, which was maintained during the whole process, 6 g of KMnO_4_ was added slowly to the round flask. Afterwards, the ice bath was removed and the system was maintained under magnetic stirring for 180 minutes. After this time, 92 mL of water was added slowly and then 280 mL of hot water was added. To the consumption of oxidize agent, 10 mL of H_2_O_2_ (30% v v^−1^) were slowly added and kept 30 minutes under stirring. Next, the mixture was transfer to 1 L beaker and it was added 500 mL of a HCl solution (10% v v^−1^). The system was magnetic stirred for 10 minutes and it was left to sediment. Finally, the solid was washed with deionized water until neutral pH, filtrated and dried at 60 °C for 24 hours generating the graphite oxide (Gr-O). 150 mg of Gr-O was exfoliated in 50 mL of deionized water using a tip ultrasound for 10 min (Cole Parmer CP505 - 20 kHz–200 W). This mixture was centrifuged for 90 minutes at 3000 rpm to remove the not dispersed material, leading to the **GO** dispersion. After that, the water was removed from the **GO** dispersion by heating at 80 °C, and the resulting solid (**GO**) remained drying at this temperature overnight.

To the synthesis of the nanocomposites, 5 mg of **GO** was dispersed in 20 mL of ethylene glycol in a 250 mL round flask using a bath ultrasound (Unique USC 1880 - 37 kHz) for 60 minutes. Next, nickel acetate was added to the system leading to the GO/Ni^2+^ weight ratios of 1/0.1 (starting from 2.2 mg of nickel acetate, yielding the sample **rGONi(OH)**_**2**_**-1**), 1/0.25 (5.5 mg of nickel acetate, sample **rGONi(OH)**_**2**_**-2**), 1/0.5 (11.1 mg of nickel acetate, sample **rGONi(OH)**_**2**_**-3**) and 1/1 (22.1 mg of nickel acetate, sample **rGONi(OH)**_**2**_**-4**). Then, a condenser was adapted to the system, which was maintained at vigorous magnetic stirring and heated for 2 hours at 190 °C. After this time, the solid material was filtrated, washed with 200 mL of deionized water and dried at 70 °C for 2 hours. Other two control samples were prepared starting from just **GO** (producing the sample **rGO**) or the nickel precursor (sample **Ni(OH)**_**2**_), where the amount of nickel acetate used was the same that in the sample **rGONi(OH)**_**2**_**-4**.

To the processing of the materials as thin films, 1 mg of each material was dispersed in 40 mL of deionized water in a 100 mL round flask using a bath ultrasound for 60 minutes. Next, 40 mL of toluene was added and the system was kept under magnetic stirring (2500 rpm) for 20 hours. After this time, the magnetic stirring was interrupted and a thin film was spontaneously deposited at the water/toluene interface. The entire round flask content was transferred with a micropipette to a 100 mL beaker containing the desired substrates. As the thin film is self-assembled, it reorganizes itself at the liquid-liquid interface. So, the substrates were pulled from the bottom of the beaker to the interface direction, aiming a homogeneous deposition of the film over them. Finally, the thin films over the substrates were dried at 100 °C for 60 minutes. All the steps involved in the thin film preparation are illustrated in the Supplementary Fig. S20.

Thermogravimetric analyses (TGA) were carried out in a TGA/SDT Q 600, using a heating rate of 5 °C min^−1^ in air atmosphere.

X-ray diffraction patterns were obtaining using a Shimadzu XRD-6000 diffractometer with Cu-Kα radiation (λ = 1.5418 Å) operating at 40 kV and 30 mA. The thin films were analyzed using a low angle accessory with 0.1° incident angle operating at 40 kV and 40 mA.

FT–IR spectra of the samples were recorded using a FT-IR Bomem spectrophotometer with 32 scans and 4 cm^−1^ of resolution. The samples were prepared in KBr pellets.

UV-Vis data of the thin films over quartz substrates were obtaining using a Shimadzu UV-2450 spectrophotometer. The UV-Vis spectroelectrochemical measurements were carried out in the same equipment using a home-made electrochemical cell.

Raman spectra were obtaining in a Renishaw Raman Imaging Microprobe System 3000 spectrophotometer using a 514.5 nm (Ar^+^ laser) excitation line.

TEM measurements were performed in a JEOL JEM 120 kV instrument. For the analysis, one drop of the samples dispersed in deionized water was evaporated onto carbon films supported on holey carbon copper grids.

SEM images were recorded using a MIRA-3 FEG-SEM Tescan with 10 kV voltage and a secondary electrons detector. The samples were analyzed either as powders sprinkled on Cu adhesive tape or as the thin films deposited over ITO substrates. EDS spectra were obtaining in the same equipment using an Oxford accessory.

The thicknesses of the films were analyzed using a Dektak 150 Veeco surface profilometer with 1 nm resolution.

Resistivity measurements of the films were carried out using a four-point Jandel Universal Probe, directly from the thin films over glass substrates.

The mass of the thin films was evaluated by measuring the mass of four layers deposited onto the same substrate with an area of 7.5 cm^2^. After each deposition the substrates were dried at 100 °C for 60 minutes under low pressure. The mass found for one thin film layer was 8.5 μg cm^−2^.

Cyclic voltammetry measurements were carried out in a Autolab potentiostat in a conventional three-electrode cell, where the thin films deposited over ITO, with a 1 cm^2^ electroactive area, were used as working electrodes, an Ag/AgCl (3 mol L^−1^ KCl) as the reference electrode and a platinum wire as the counter electrode. The cyclic voltammetry for the lithium intercalation test was carried out in 1 mol L^−1^ LiClO_4_ (Vetec) propylenecarbonate (Aldrich) solution using a scan rate of 1 mV s^−1^. The UV-Vis spectroelectrochemical measurements were carried out with the films deposited over ITO with a 1.5 cm^2^ electroactive area. The charge-discharge measurements were performed using cut-off potentials of 0 and 0.5 V and 3 cycles for each charge-discharge current. The charge-discharge currents were calculated using the whole mass of the film.

The chronoamperograms for the glycerol detection were recorded under magnetic stirring (1100 rpm). The EIS data were obtaining using the NOVA software. For that, the thin films were first submitted to 150 voltammetric cycles at 50 mV s^−1^ and then a potential of 0.39 V was applied for 5 minutes. Next, impedance analyses were carried out at frequencies ranging from 10^−1^ to 10^4^ Hz, using a standard potential of 0.39 V and an amplitude potential of 10 mV. These analyses were also performed after the 2000 cycles of charge-discharge. The data were fitted using the ZView software. All the electrochemical measurements were carried out in 1 mol L^−1^ NaOH aqueous solution.

## Additional Information

**How to cite this article**: Neiva, E. G. C. *et al.* One material, multiple functions: graphene/Ni(OH)_2_ thin films applied in batteries, electrochromism and sensors. *Sci. Rep.* **6**, 33806; doi: 10.1038/srep33806 (2016).

## Supplementary Material

Supplementary Information
