# Supplementary material for: One material, multiple functions: graphene/Ni(OH)2 thin films applied in batteries, electrochromism and sensors
Source: Sci Rep. 2016 Sep 22;6:33806. doi: 10.1038/srep33806 (PMC5031963; doi:10.1038/srep33806)
Supplement: Supplementary Information [file srep33806-s1.docx]

**SUPPLEMENTARY MATERIAL**

*One material, multiple functions: graphene/Ni(OH)_2_ thin films applied in batteries, electrochromism and sensors*

*Eduardo G. C. Neiva^a^, Marcela M. Oliveira^b^, Márcio F. Bergamini^a^, Luiz H. Marcolino Jr.^a^ and Aldo J. G. Zarbin^a,*^*

^a^Departamento de Química, Universidade Federal do Paraná (UFPR), CP 19081, CEP 81531-990, Curitiba, PR, Brazil.

^b^Departamento de Química e Biologia, Universidade Tecnológica Federal do Paraná (UTFPR), Curitiba, PR, Brazil.

**
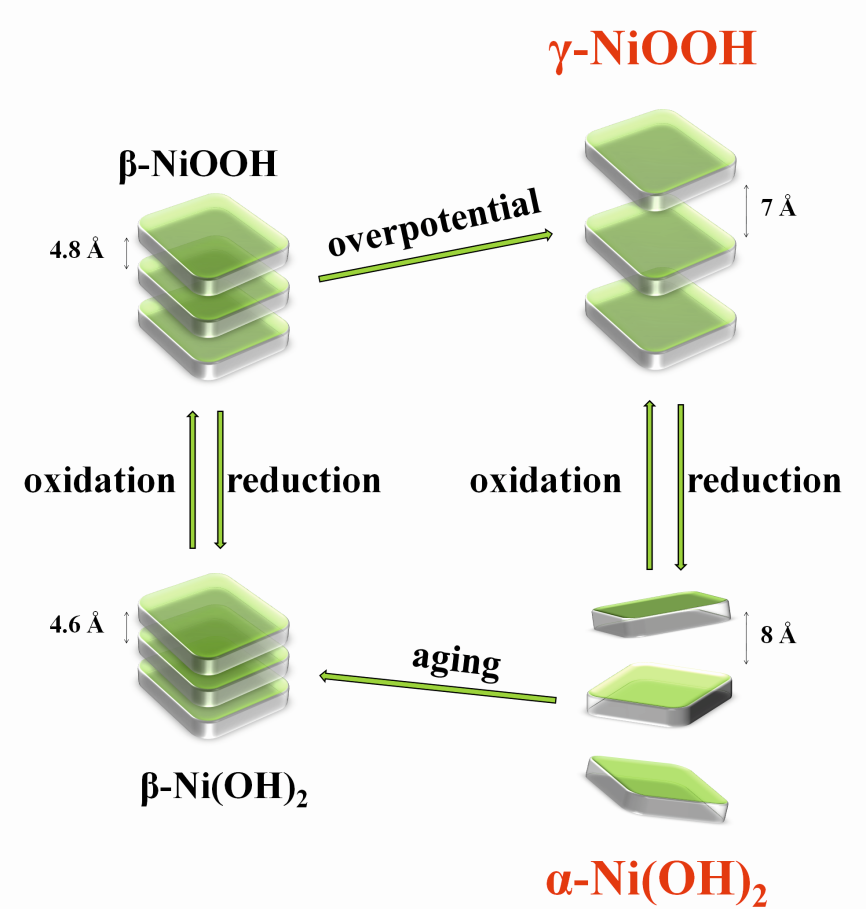
**

**Figure S1.** Bode diagram correlating the Ni(OH)_2_ and NiOOH structures.^1^


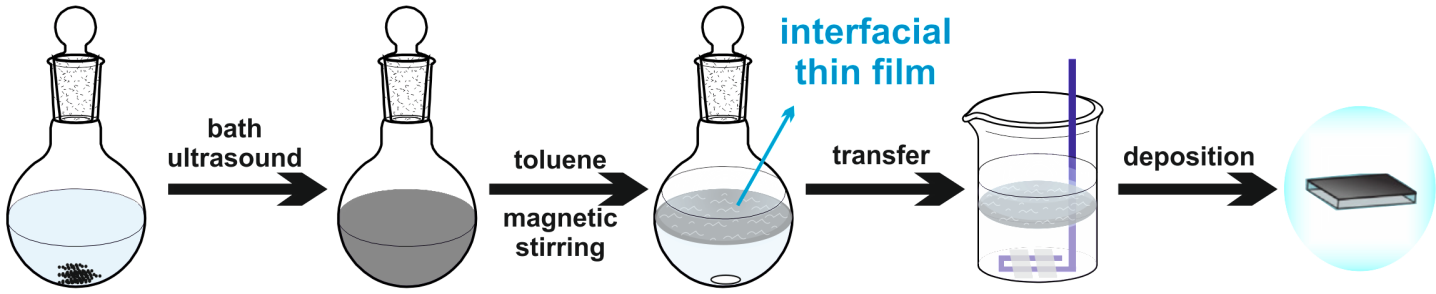


**Figure S2.** Illustration of the thin film preparation using the interfacial system.


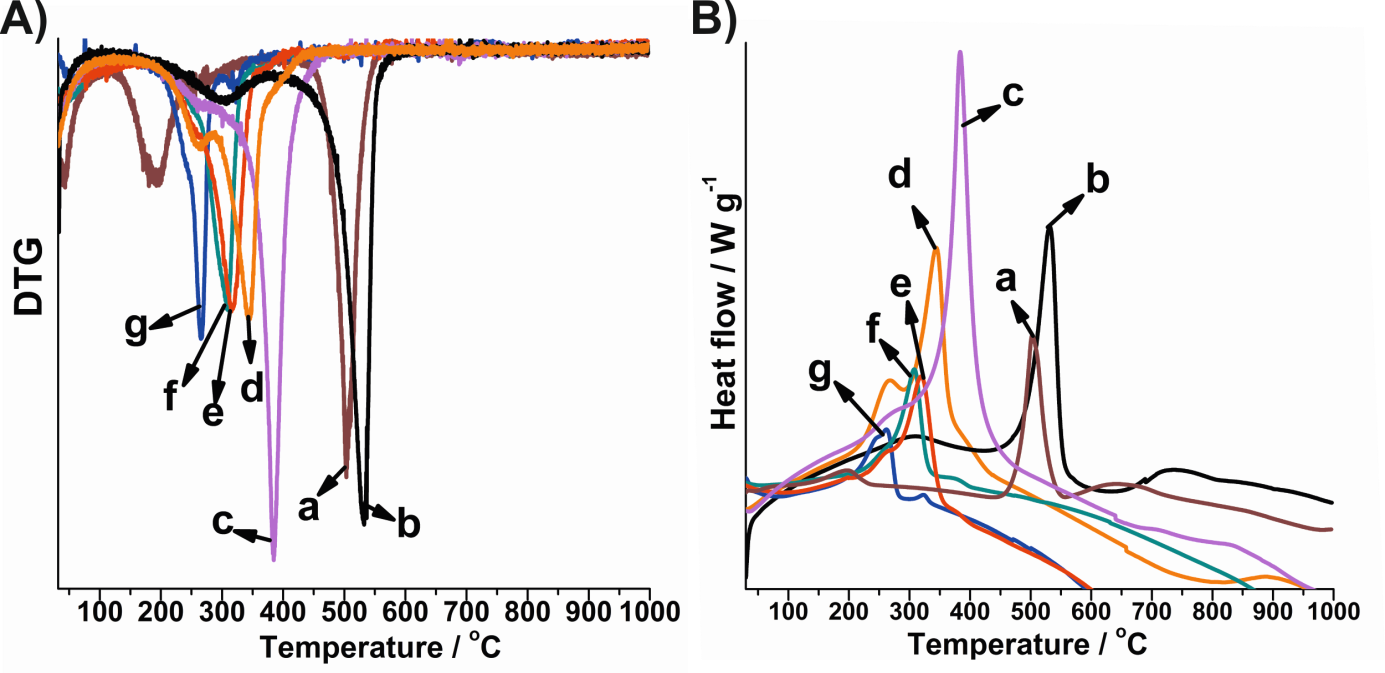


**Figure S3.** (A) DTG and (B) DSC curves of GO (a), rGO (b), rGONi(OH)_2_-1 (c), rGONi(OH)_2_-2 (d), rGONi(OH)_2_-3 (e), rGONi(OH)_2_-4 (f) and Ni(OH)_2_ (g) in air atmosphere.


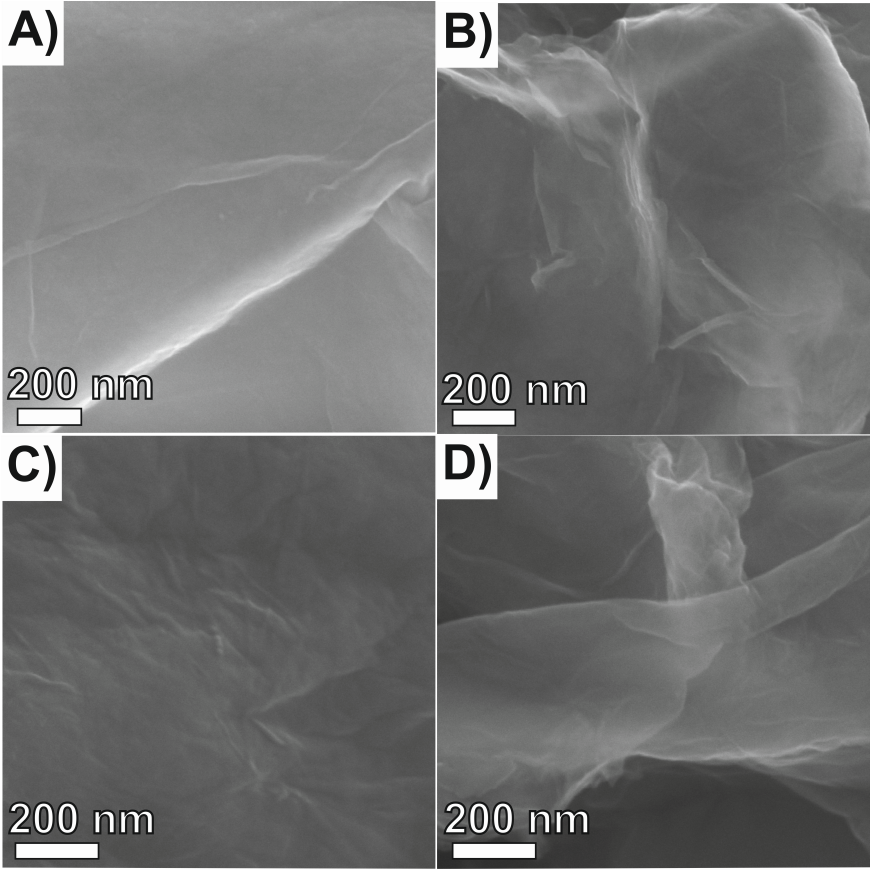


**Figure S4.** (A) FEG-SEM images of rGONi(OH)_2_-3, (B) rGONi(OH)_2_-2, (C) rGONi(OH)_2_-1 and (D) rGO.


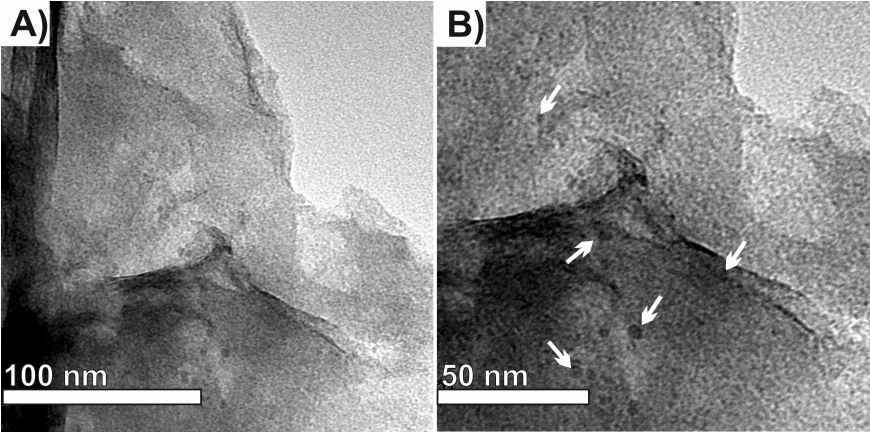


**Figure S5.** TEM images of rGONi(OH)_2_-3.


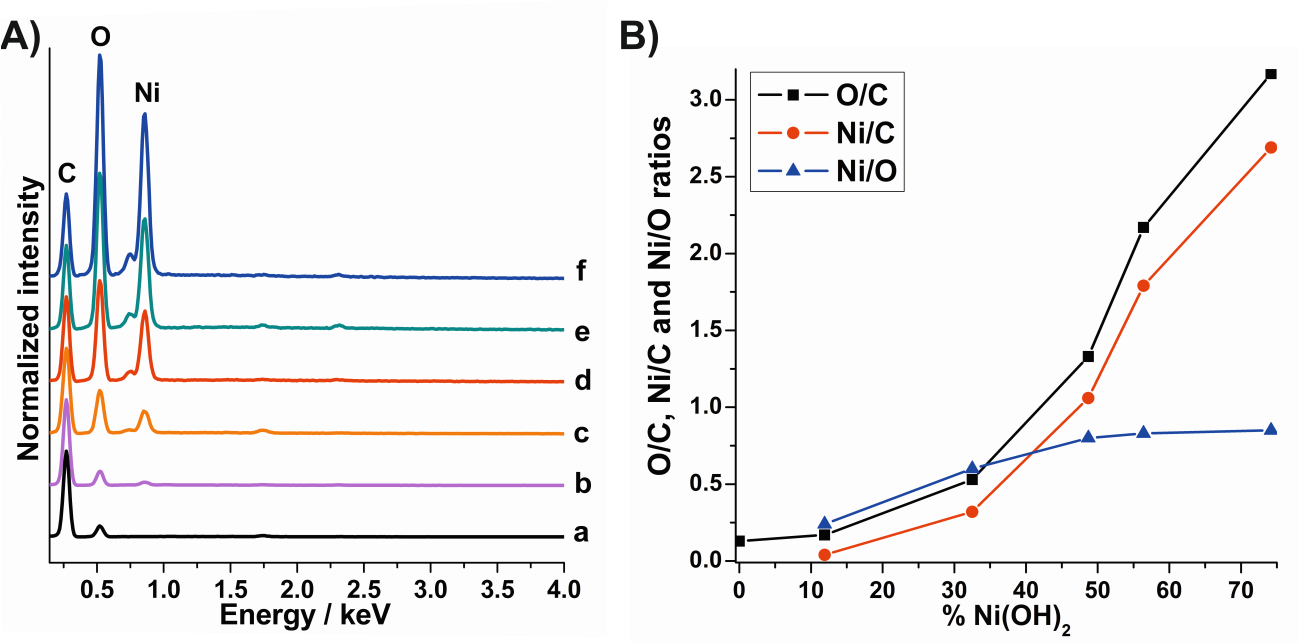


**Figure S6.** (A) EDS spectra normalized by the carbon peak of rGO (a), rGONi(OH)_2_-1 (b), rGONi(OH)_2_-2 (c), rGONi(OH)_2_-3 (d), rGONi(OH)_2_-4 (e) and Ni(OH)_2_ (f). (B) Oxygen/carbon, nickel/carbon and nickel/oxygen peak area ratios as function of Ni(OH)_2_ percentage for the nanocomposites and rGO and Ni(OH)_2_ control samples.


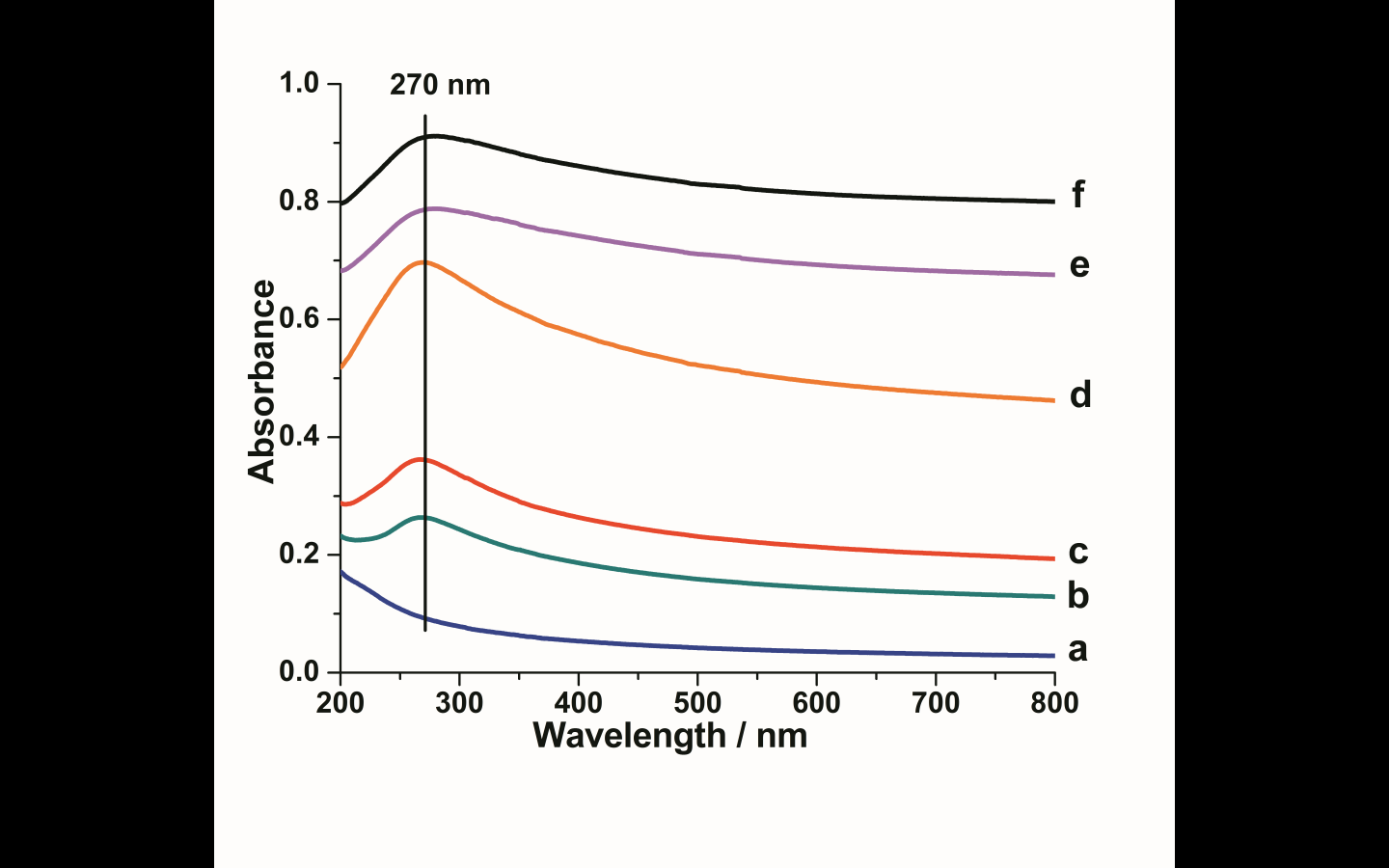


**Figure S7.** UV-Vis spectra of Ni(OH)_2_ (a), rGONi(OH)_2_-4 (b), rGONi(OH)_2_-3 (c), rGONi(OH)_2_-2 (d), rGONi(OH)_2_-1 (e) and rGO (f) thin films over quartz substrates.

**
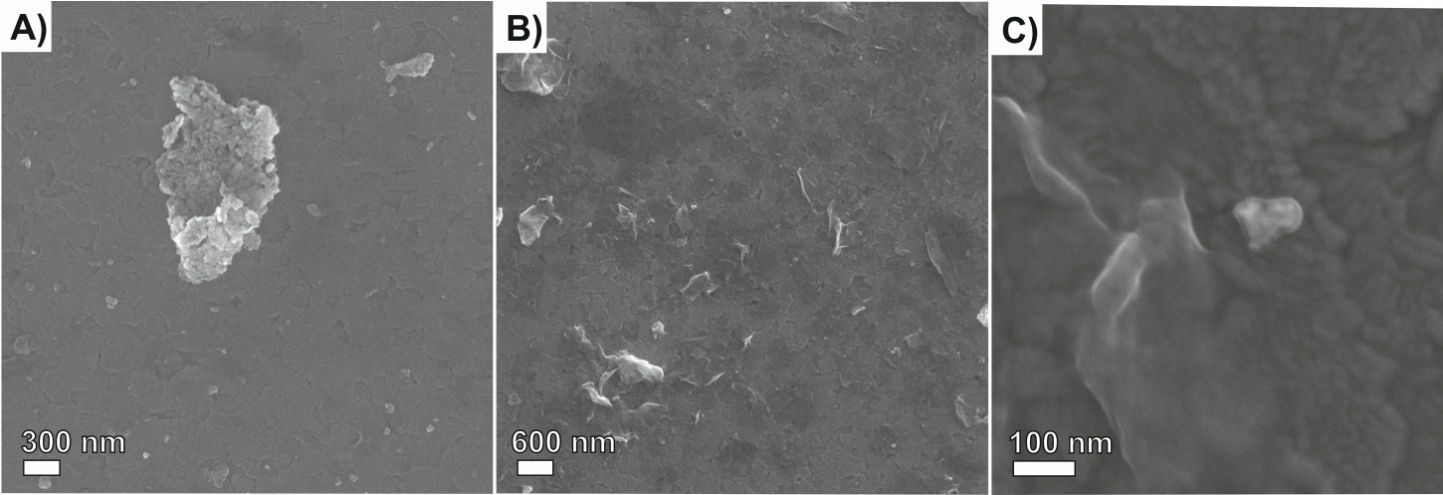
**

**Figure S8.** (A) FEG-SEM images of Ni(OH)_2_ and (B, C) rGONi(OH)_2_-4 thin films over ITO substrates.


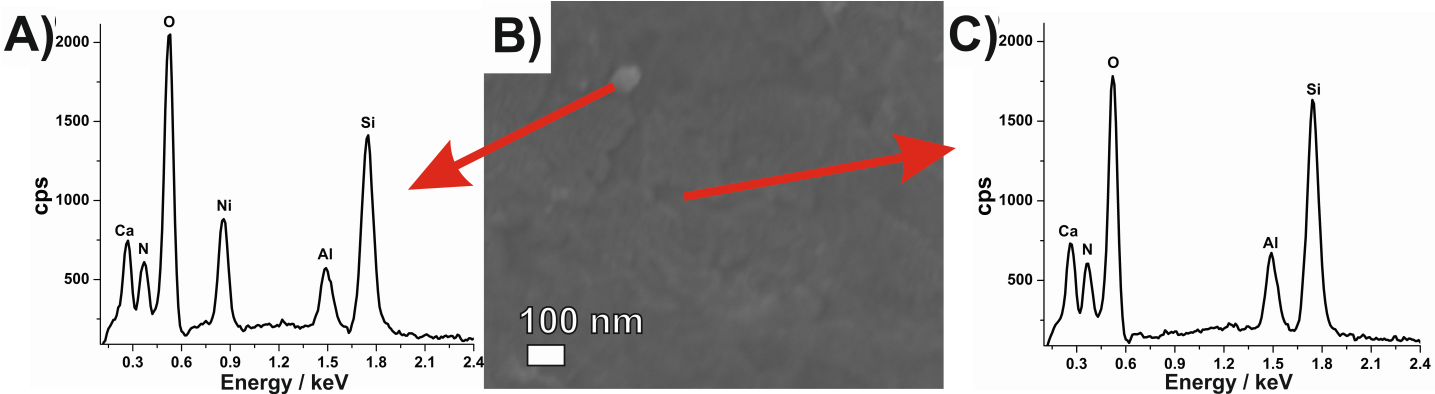


**Figure S9.** (A, C) Point EDS spectra and (B) FEG-SEM image of rGONi(OH)_2_-4 thin film over ITO substrate.


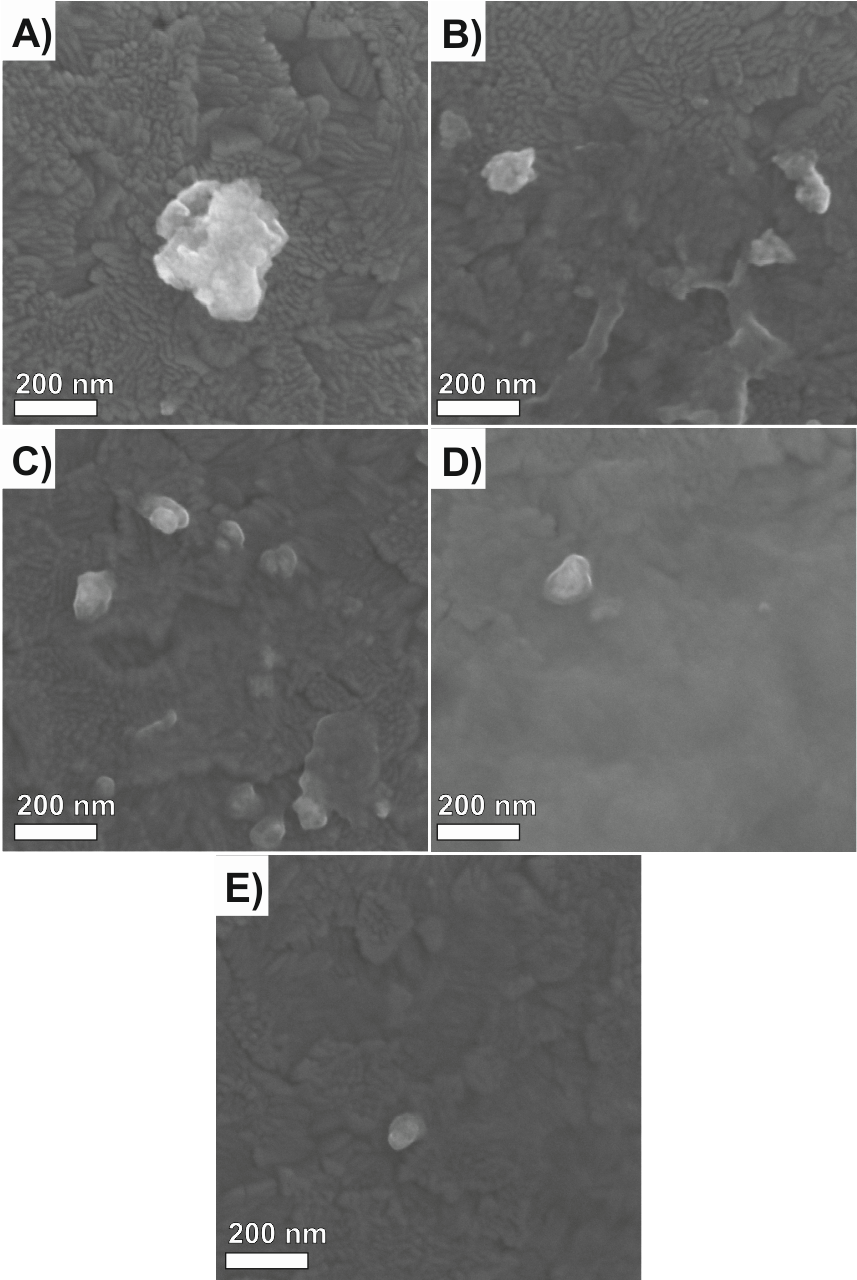


**Figura S10.** (A) FEG-SEM images of Ni(OH)_2_, (B) rGONi(OH)_2_-4, (C) rGONi(OH)_2_-3, (D) rGONi(OH)_2_-2 and (E) rGONi(OH)_2_-1 thin films over ITO after 150 voltammetric cycles.


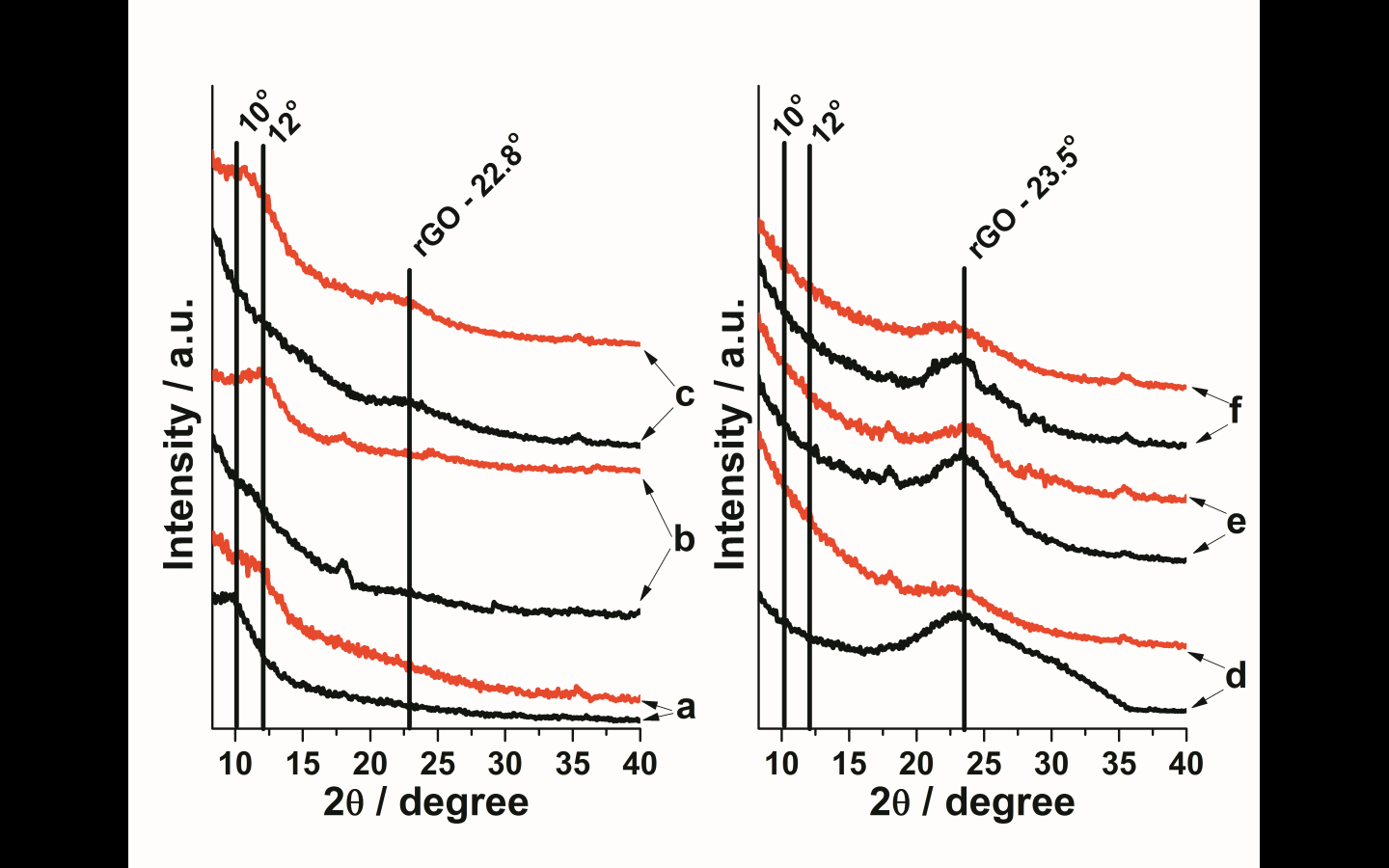


**Figure S11.** X-ray diffractograms of Ni(OH)_2_ (a), rGONi(OH)_2_-4 (b), rGONi(OH)_2_-3 (c), rGONi(OH)_2_-2 (d), rGONi(OH)_2_-1 (e) and rGO (f) thin films over ITO substrates before (▬) and after (▬) 150 voltammetric cycles in 1 mol L^-1^ NaOH. It was used a low angle accessory with 0.1º incident angle.


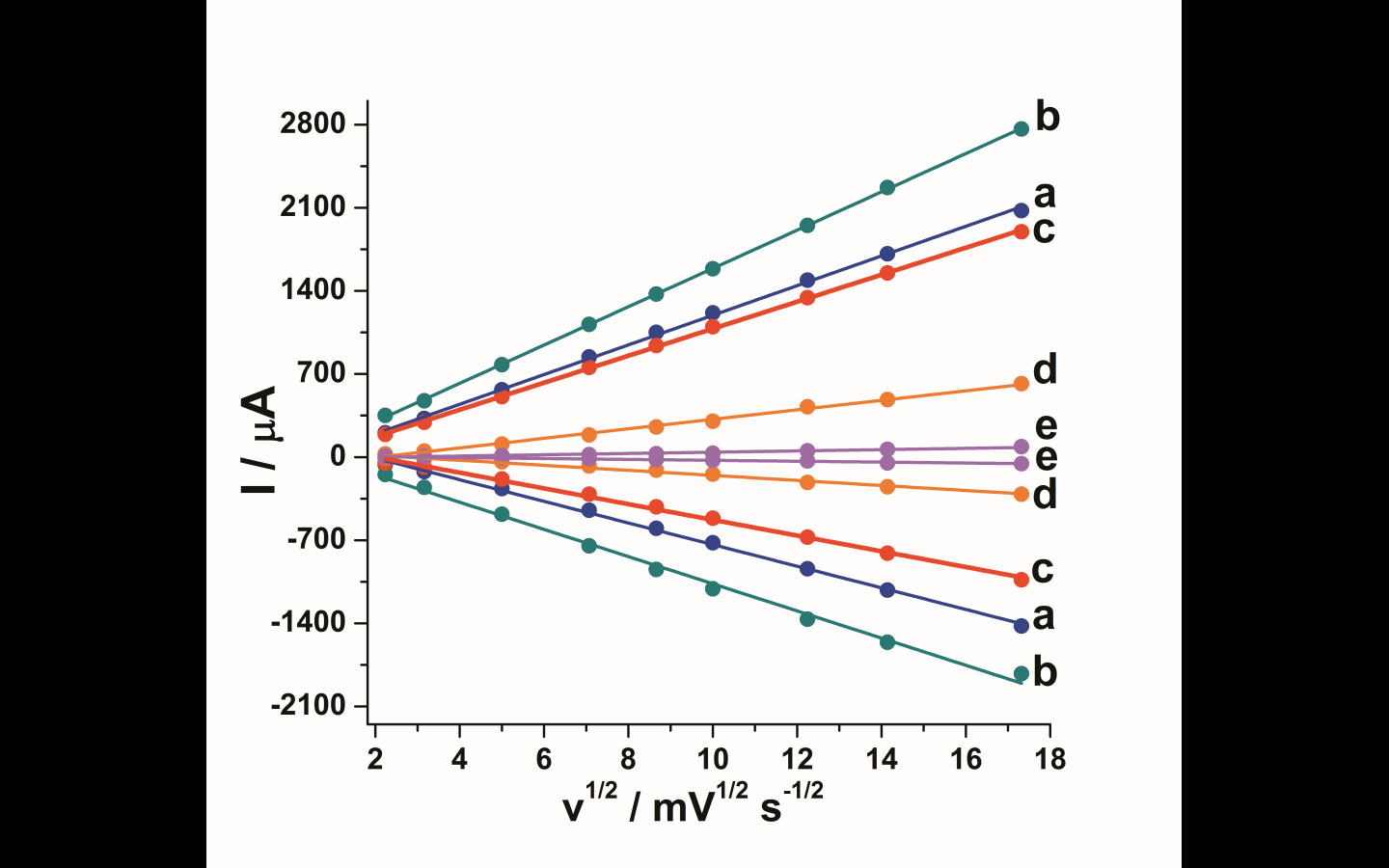


**Figure S12.** Anodic and cathodic current peak as function of the v^1/2^ of the Ni(OH)_2_ (a), rGONi(OH)_2_-4 (b), rGONi(OH)_2_-3 (c), rGONi(OH)_2_-2 (d) and rGONi(OH)_2_-1 (e) thin films.


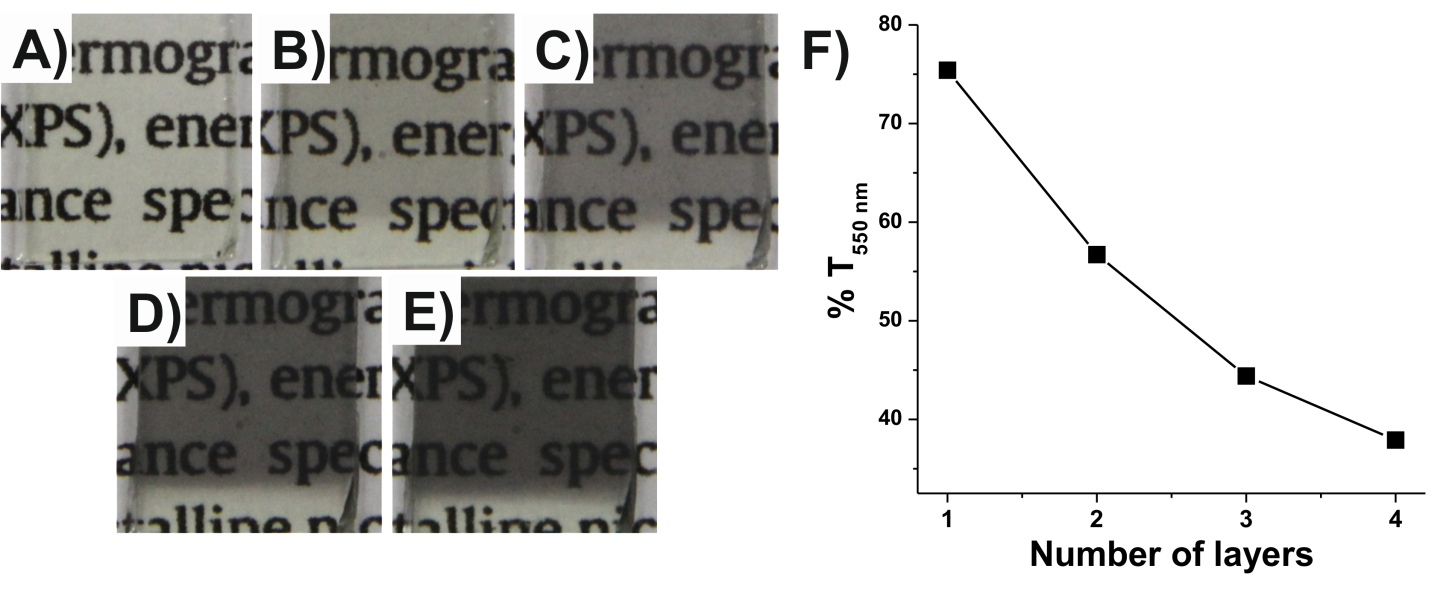


**Figure S13.** (A) Photographic images of ITO and (B) rGONi(OH)_2_-4 thin films with one, (C) two, (D) three and (E) four layers. Transmittance at 550 nm as function of the number of layers.


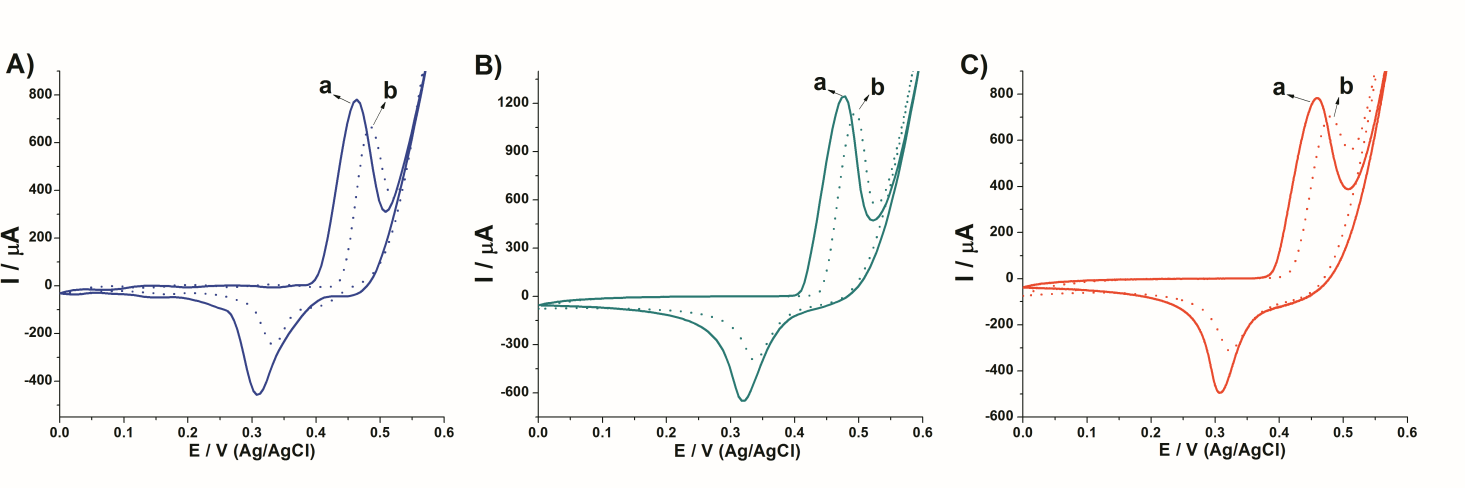


**Figure S14.** (A) Voltammograms of Ni(OH)_2_, (B) rGONi(OH)_2_-4 and (C) rGONi(OH)_2_-3 thin films before (a) and after 2000 charge-discharge cycles (b).

**
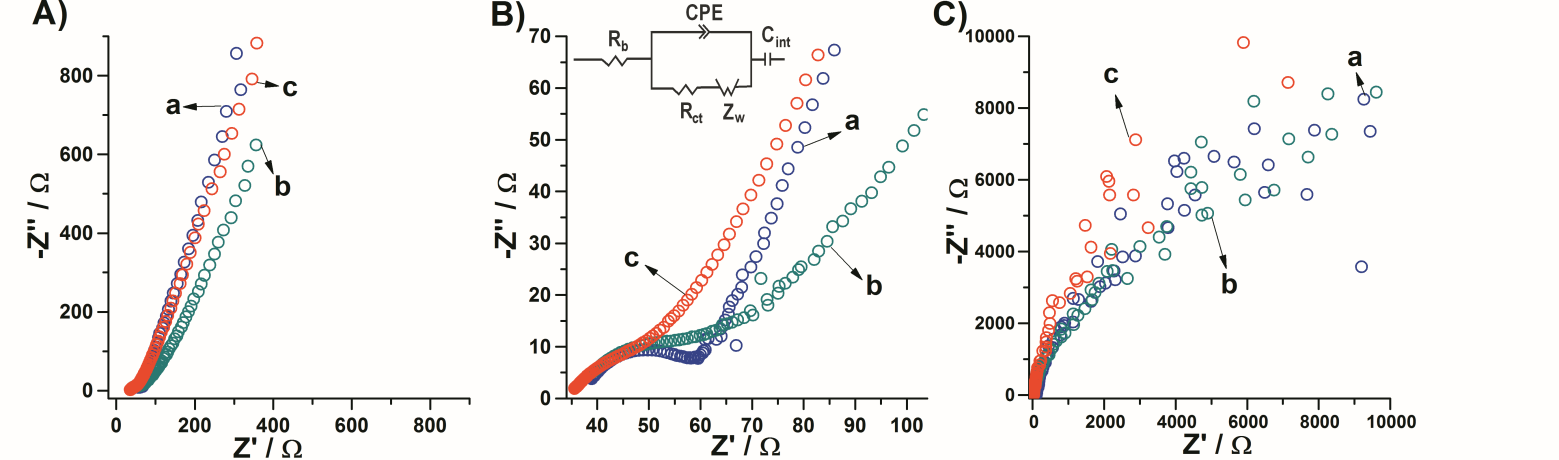
**

**Figure S15.** (A, B) EIS spectra before and (C) after 2000 charge-discharge cycles of Ni(OH)_2_ (a), rGONi(OH)_2_-4 (b) and rGONi(OH)_2_-3 (c) thin films. The inset in (B) is the equivalent circuit adopted. It was used a potential of 0.39 V, a frequency range from 10^-1^ to 10^4^ Hz and an amplitude potential of 10 mV.


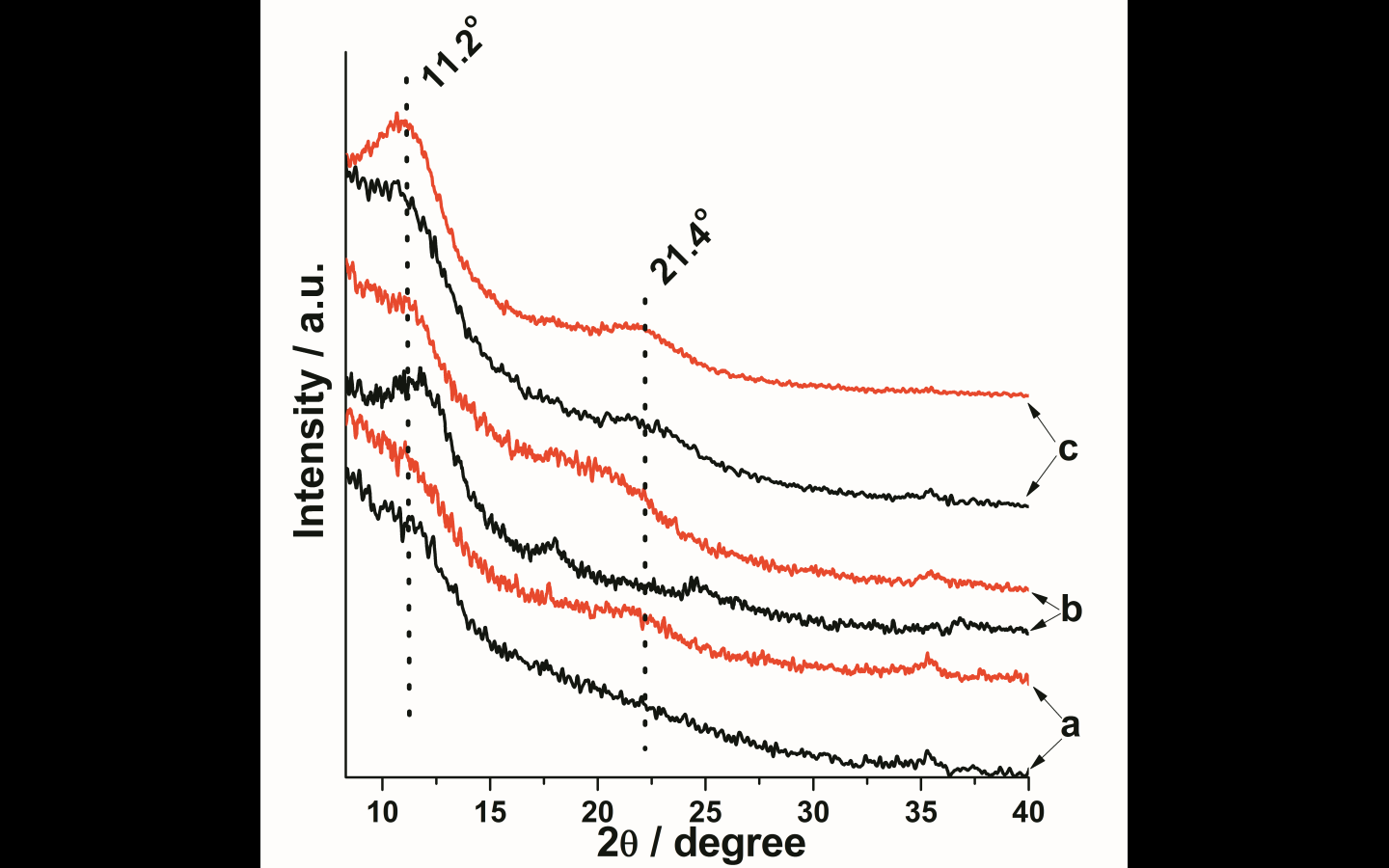


**Figure S16.** X-ray diffractograms of Ni(OH)_2_ (a), rGONi(OH)_2_-4 (b) and rGONi(OH)_2_-3 (c) thin films over ITO substrates before (▬) and after (▬) 2000 charge-discharge cycles. It was used a low angle accessory with 0.1º incident angle.

**
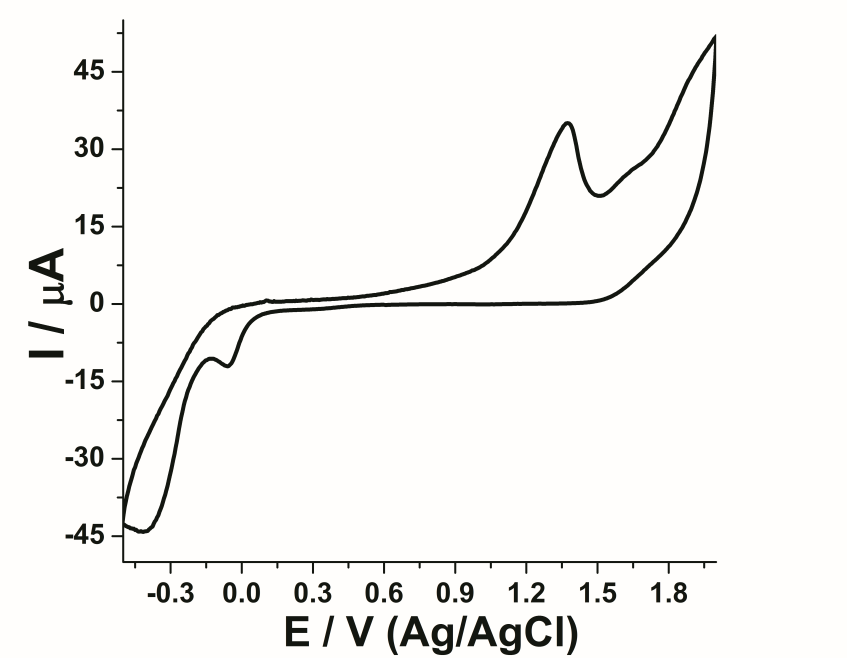
**

**Figure S17.**  Second cyclic voltammogram of rGONi(OH)_2_-4 in 1 mol L^-1^ LiClO_4_/propylene carbonate solution using a scan rate of 1 mV s^-1^.


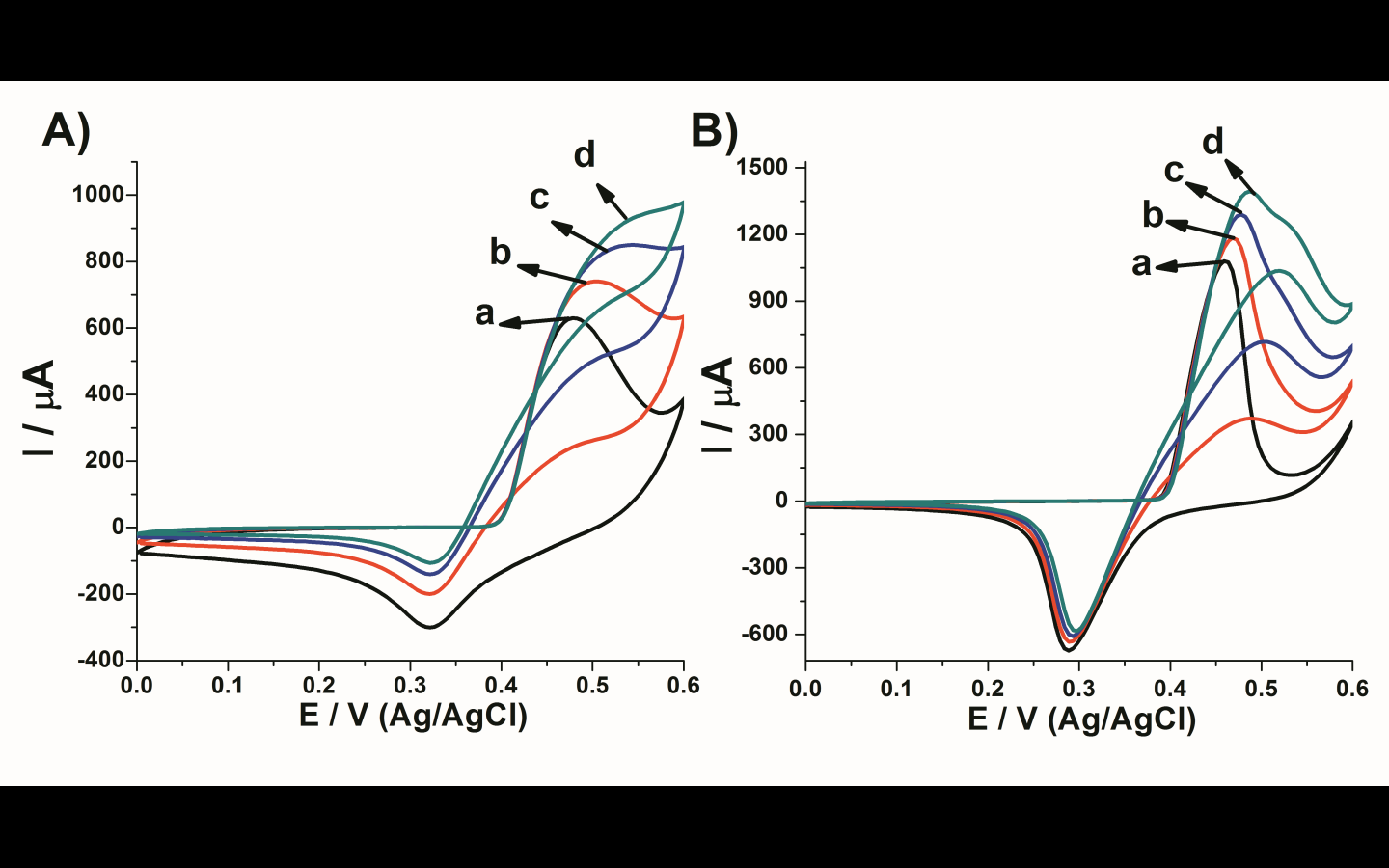


**Figure S18.** (A) Voltammograms of rGONi(OH)_2_-3 and (B) Ni(OH)_2_ thin films in the absence (a) and presence of 1 (b), 2 (c) and 3 mmol L^-1^ glycerol (d) in 1 mol L^-1^ NaOH at 50 mV s^-1^.


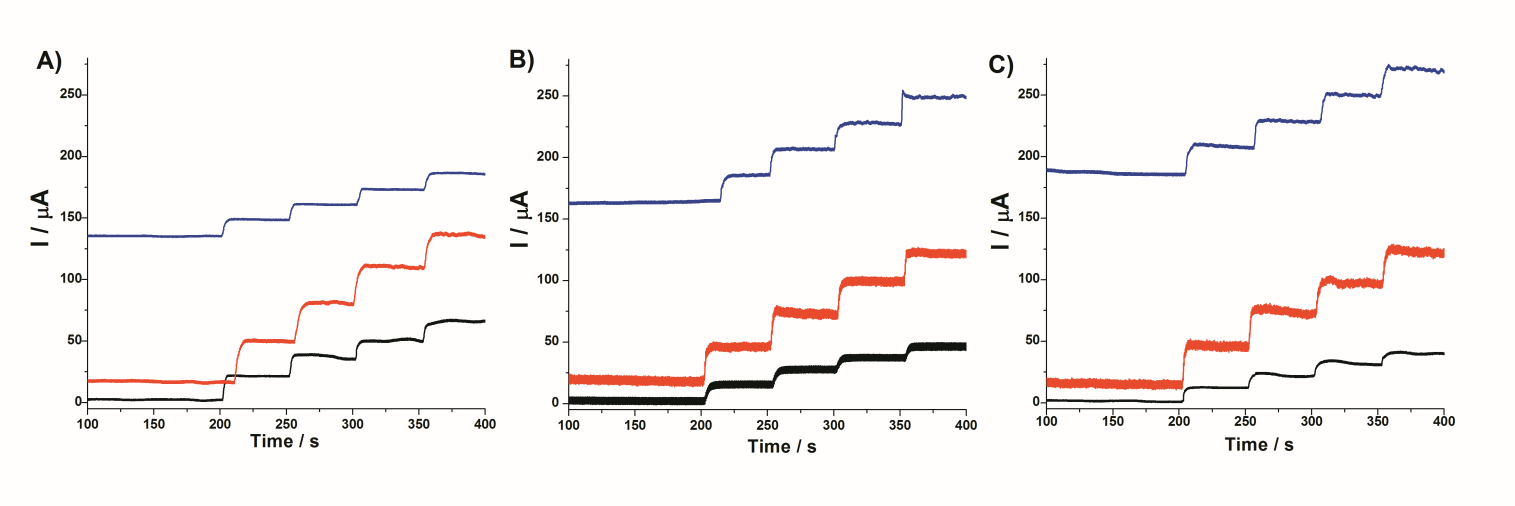


**Figure S19.** (A) Chronoamperograms of rGONi(OH)_2_-4, (B) rGONi(OH)_2_-3 and (C) Ni(OH)_2_ thin films with 4 additions of 100 μmol L^-1^ glycerol using the potentials of 0.4 V (a), 0.45 (b) and 0.5 V (c) in 1 mol L^-1^ NaOH.


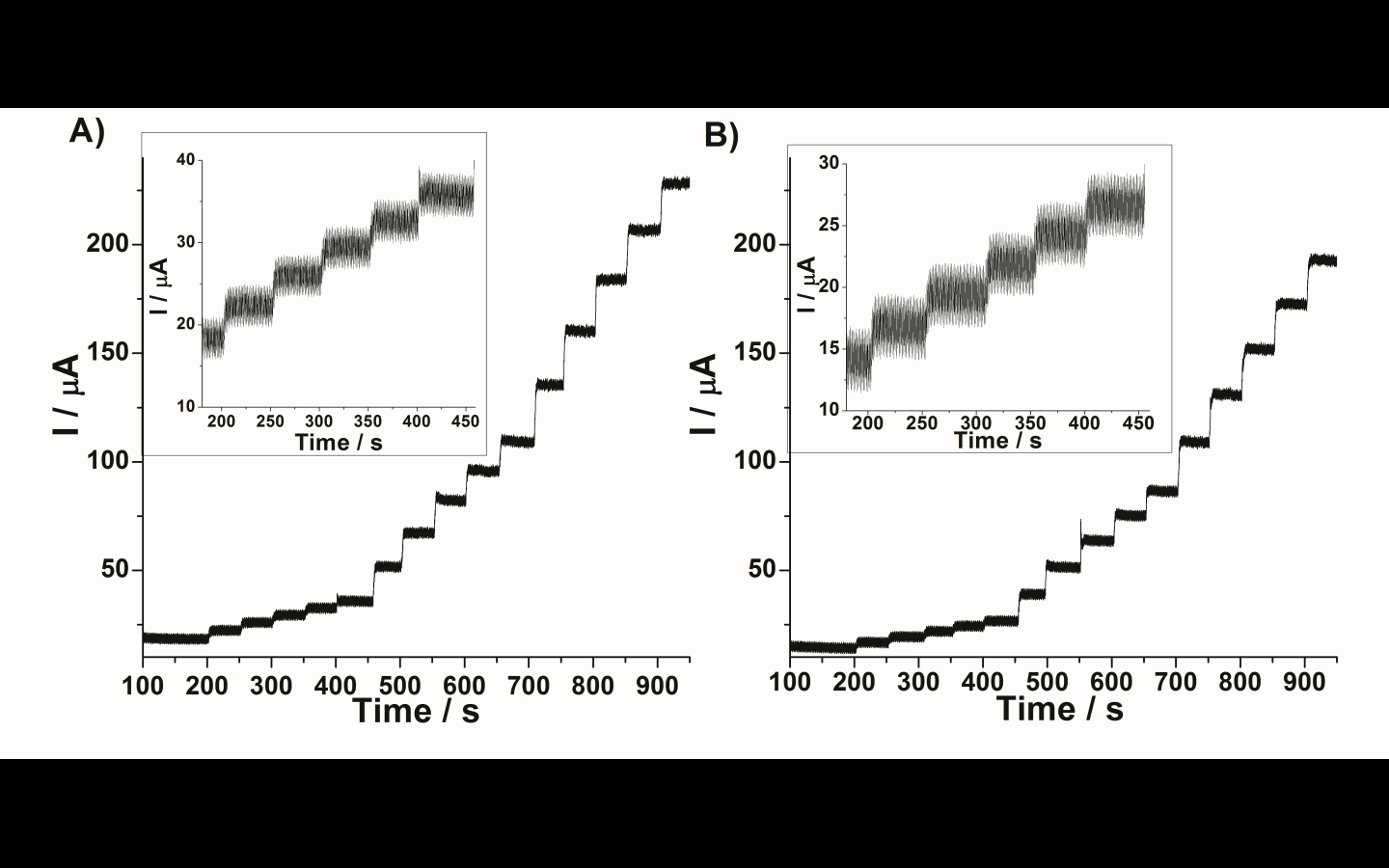


**Figure S20.** (A) Chronoamperograms for rGONi(OH)_2_-3 and (B) Ni(OH)_2_ thin films with glycerol additions from 10 to 800 μmol L^-1^ in 1 mol L^-1^ NaOH.

**Table S1.** Loss mass events percentages from 20 to 120 ºC and from 130 to 580 ºC, and NiO and Ni(OH)_2_ percentages. Decomposition temperature of Ni(OH)_2_ (T_Ni(OH)2_), GO and rGO (T_C_) from DTG.

|  | %_20 to 120 ºC_ | %_130 to 580 ºC_ | % NiO | % Ni(OH)_2_ | T_Ni(OH)2_ | T_C_ |
| --- | --- | --- | --- | --- | --- | --- |
| Ni(OH)_2_ | 5.4 | 38.4 | 59.8 | 74.2 | 266 | --- |
| rGONi(OH)_2_-4 | 11.5 | 43.2 | 45.5 | 56.4 | 254 | 309 |
| rGONi(OH)_2_-3 | 11.5 | 52.5 | 39.2 | 48.7 | 260 | 317 |
| rGONi(OH)_2_-2 | 12.9 | 62 | 26.2 | 32.5 | 263 | 344 |
| rGONi(OH)_2_-1 | 8.8 | 83 | 9.6 | 11.9 | 268 | 385 |
| rGO | 6.7 | 88.3 | --- | --- | --- | 532 |
| GO | 15.6 | 82.4 | --- | --- | --- | 505 |

**Table S2.** Analysis system, linear detection range and LOD for the thin films developed in this report compared with the literature.

| Electrode | Analysis system | Linear detection range | LOD | Ref. |
| --- | --- | --- | --- | --- |
| rGONi(OH)_2_-3-modified ITO electrode | chronoamperometry | 10-800 μmol L^-1^ | 15.4 µmol L^-1^ | - |
| rGONi(OH)_2_-4-modified ITO electrode | chronoamperometry | 10-800 μmol L^-1^ | 25 µmol L^-1^ | - |
| Ni(OH)_2_-modified ITO electrode | chronoamperometry | 10-800 μmol L^-1^ | 20.2 µmol L^-1^ | - |
| boron-doped diamond electrode | chronoamperometry | 11-1400 µmol L^-1^ | 11 µmol L^-1^ | 2 |
| Au (111)/SiO_2_ cavity/ ITO electrode | DPV | 10-800 µmol L^-1^ | 1.49 µmol L^-1^ | 3 |
| Platinum electrode | potential cycling technique | 160-1600 µmol L^-1^ | 25 µmol L^-1^ | 4 |
| Ni nanoparticles-modified carbon paste electrode | chronoamperometry | 95-8000 µmol L^-1^ | 95 µmol L^-1^ | 5 |
| glycerol oxidase-based biosensor | chronoamperometry | 50-25600 µmol L^-1^ | 50 µmol L^-1^ | 6 |
| nanonickel modified graphite electrode | DPV | 500-12000 µmol L^-1^ | 33 µmol L^-1^ | 7 |
| hcp-Ni thin film | chronoamperometry | 1-500 µmol L^-1^ | 2.4 µmol L^-1^ | 8 |

**REFERENCES**

1 Bode, H., Dehmelt, K. & Witte, J. Zur kenntnis der nickelhydroxidelektrode--I. Über das nickel (II)-hydroxidhydrat. *Electrochim. Acta* **11**, 1079-1087 (1966).

2 Pop, A. *et al.* Non-enzymatic electrochemical detection of glycerol on boron-doped diamond electrode. *Analyst* **137**, 641-647 (2012).

3 Li, N. *et al.* Electrochemical detection of free glycerol in biodiesel using electrodes with single gold particles in highly ordered SiO2 cavities. *Sens. Actuators B Chem.* **196**, 314-320 (2014).

4 Lourenço, L. M. & Stradiotto, N. R. Determination of free glycerol in biodiesel at a platinum oxide surface using potential cycling technique. *Talanta* **79**, 92-96 (2009).

5 Neiva, E. G. C., Bergamini, M. F., Oliveira, M. M., Marcolino Jr, L. H. & Zarbin, A. J. G. PVP-capped nickel nanoparticles: Synthesis, characterization and utilization as a glycerol electrosensor. *Sens. Actuators B Chem.* **196**, 574-581 (2014).

6 Goriushkina, T. B. *et al.* Amperometric biosensor based on glycerol oxidase for glycerol determination. *Sens. Actuators B Chem.* **144**, 361-367 (2010).

7 M.A. Tehrani, R. & Ab Ghani, S. Electrocatalysis of free glycerol at a nanonickel modified graphite electrode and its determination in biodiesel. *Electrochim. Acta* **70**, 153-157 (2012).

8 Neiva, E. G. C., Oliveira, M. M., Marcolino Jr, L. H. & Zarbin, A. J. G. Nickel nanoparticles with hcp structure: Preparation, deposition as thin films and application as electrochemical sensor. *J. Colloid Interface Sci.* **468**, 34-41 (2016).
